# RNAmountAlign: Efficient software for local, global, semiglobal pairwise and multiple RNA sequence/structure alignment

**DOI:** 10.1371/journal.pone.0227177

**Published:** 2020-01-24

**Authors:** Amir H. Bayegan, Peter Clote

**Affiliations:** Biology Department, Boston College, Chestnut Hill, MA, United States of America; University of Michigan, UNITED STATES

## Abstract

Alignment of structural RNAs is an important problem with a wide range of applications. Since function is often determined by molecular structure, RNA alignment programs should take into account both sequence and base-pairing information for structural homology identification. This paper describes C++ software, RNAmountAlign, for RNA sequence/structure alignment that runs in *O*(*n*^3^) time and *O*(*n*^2^) space for two sequences of length *n*; moreover, our software returns a *p*-value (transformable to expect value *E*) based on Karlin-Altschul statistics for local alignment, as well as parameter fitting for local and global alignment. Using incremental mountain height, a representation of structural information computable in cubic time, RNAmountAlign implements quadratic time pairwise local, global and global/semiglobal (query search) alignment using a weighted combination of sequence and structural similarity. RNAmountAlign is capable of performing progressive multiple alignment as well. Benchmarking of RNAmountAlign against LocARNA, LARA, FOLDALIGN, DYNALIGN, STRAL, MXSCARNA, and MUSCLE shows that RNAmountAlign has reasonably good accuracy and faster run time supporting all alignment types. Additionally, our extension of RNAmountAlign, called RNAmountAlignScan, which scans a target genome sequence to find hits having high sequence and structural similarity to a given query sequence, outperforms RSEARCH and sequence-only query scans and runs faster than FOLDALIGN query scan.

## Introduction

A number of different metrics exist for comparison of RNA secondary structures, including base pair distance (BP), string edit distance (SE) [1], mountain distance (MD) [2], tree edit distance (TE) [3], coarse tree edit distance (HTE) [4], morphological distance [5] and a few other metrics. In what appears to be the most comprehensive published comparison of various secondary structure metrics [6], it was shown that all of these distance measures are highly correlated with respect to Pearson correlation when computing distances between structures taken from the Boltzmann low-energy ensemble of secondary structures [7] for the same RNA sequence—so-called *intra-ensemble* correlation. In contrast, these distance measures have low Pearson correlation when computing distances between structures taken from Boltzmann ensembles of different RNA sequences of the same length—so-called *inter-ensemble* correlation. For instance, the intra-ensemble correlation between base pair distance (BP) and mountain distance (MD) is 0.822, while the corresponding inter-ensemble correlation drops to 0.210. Intra-ensemble correlation between string edit distance (SE) and the computationally more expensive tree edit distance (TE) is 0.975, while the corresponding intra-ensemble correlation drops to 0.590—see Table 1.

**Table 1:** Pearson correlation between various secondary structure metrics.

	BP	MD	SE	TE	HTE
BP		0.210	0.134	0.133	0.230
MD	0.822		0.519	0.607	0.515
SE	0.960	0.853		0.590	0.310
TE	0.943	0.879	0.975		0.597
HTE	0.852	0.844	0.879	0.913	

Pearson correlation between various secondary structure metrics, as computed in [6]: base pair distance (BP), string edit distance (SE) [1], mountain distance (MD) [2], tree edit distance (TE) [3] and coarse tree edit distance (HTE) [4]. Lower triangular values indicate intra-ensemble correlations; upper triangular values indicate inter-ensemble correlations. Table values are taken from [6].

Due to poor inter-ensemble correlation of RNA secondary structure metrics, and the fact that most secondary structure pairwise alignment algorithms depend essentially on some form of base pair distance, string edit distance, or free energy of common secondary structure, we have developed the first RNA sequence/structure pairwise alignment algorithm that is based on (incremental ensemble) mountain distance. Our software, RNAmountAlign, uses this distance measure, since the Boltzmann ensemble of all secondary structures of a given RNA of length *n* can represented as a length *n* vector of real numbers, thus allowing an adaptation of fast sequence alignment methods. Depending on the command-line flag given, our software, RNAmountAlign can perform pairwise alignment, (Needleman-Wunsch global [8], Smith-Waterman local [9] or semiglobal [10] alignment) as well as progressive multiple alignment (global and local), computed using a guide tree as in CLUSTAL [11]. Expect values *E* for local alignments are computed using Karlin-Altschul extreme-value statistics [12, 13], suitably modified to account for our new sequence/structure similarity measure. Additionally, RNAmountAlign can determine *p*-values (hence *E*-values) by parameter fitting for the normal (ND), extreme value (EVD) and gamma (GD) distributions.

We benchmark the performance of RNAmountAlign on pairwise and multiple global sequence/structure alignment of RNAs against the widely used programs LARA, FOLDALIGN, DYNALIGN, LocARNA, STRAL and MXSCARNA. LARA (Lagrangian relaxed structural alignment) [14] formulates the problem of RNA (multiple) sequence/structure alignment as a problem in integer linear programming (ILP), then computes optimal or near-optimal solutions to this problem. The software FOLDALIGN [15–17], and DYNALIGN [18] are different *O*(*n*^4^) approximate implementations of Sankoff’s *O*(*n*^6^) optimal RNA sequence/structure alignment algorithm. FOLDALIGN sets limits on the maximum length of the alignment as well as the maximum distance between subsequences being aligned in order to reduce the time complexity of the Sankoff algorithm. DYNALIGN [18] implements pairwise RNA secondary structural alignment by determining the common structure to both sequences that has lowest free energy, using a positive (destabilizing) energy heuristic for gaps introduced, in addition to setting bounds on the distance between subsequences being aligned. In particular, the only contribution from nucleotide information in Dynalign is from the nucleotide-dependent free energy parameters for base stacking, dangles, etc. LocARNA (local alignment of RNA) [19, 20] is a heuristic implementation of PMcomp [21] which compares the base pairing probability matrices computed by McCaskill’s algorithm. Although the software is not maintained, STRAL [22] which is similar to our approach, uses up- and downstream base pairing probabilities as the structural information and combines them with sequence similarity in a weighted fashion. MXSCARNA [23] is a progressive multiple alignment algorithm based on the pairwise alignment algorithm of SCARNA [24]. In contrast to Sankoff-type methods, SCARNA is a heuristic algorithm that performs alignment based on the detection of fixed-length stem candidates that belong to the consensus secondary structure of given sequences.

LARA, mLocARNA (extension of LocARNA), FOLDALIGNM [16, 25] (extension of FOLDALIGN), Multilign [26, 27] (extension of DYNALIGN), STRAL and MXSCARNA support multiple alignment. LARA computes all pairwise sequence alignments and subsequently uses the T-Coffee package [28] to construct multiple alignments. Both FOLDALIGNM and mLocARNA implement progressive alignment of consensus base pairing probability matrices using a guide tree similar to the approach of PMmulti [21]. For a set of given sequences, Multilign uses DYNALIGN to compute the pairwise alignment of a single fixed index sequence to each other sequence in the set, and computes a consensus structure. In each pairwise alignment, only the index sequence base pairs found in previous computations are used. More iterations in the same manner with the same index sequence are then used to improve the structure prediction of other sequences. The number of pairwise alignments in Multilign is linear with respect to the number of sequences. STRAL and MXSCARNA perform multiple alignment in a fashion similar to CLASTALW [29]. Table 2 provides an overview of various features, to the best of our knowledge, supported by the software benchmarked in this paper.

**Table 2:** Software features.

Software	Local	Global	Semiglobal	E-value	F1(Pairwise)	SPS(Multiple)
RNAmountAlign	✓	✓	✓	✓	0.84	0.81
LocARNA	✓	✓	—	—	0.81	0.86
LARA	—	✓	—	—	0.84	0.85
FOLDALIGN	✓	✓	—	✓	0.80	0.74
DYNALIGN	—	✓	—	—	0.68	0.63
STRAL	—	✓	—	—	0.82	—
MXSCARNA	✓	✓	—	—	0.84	0.84

Overview of features in software used in benchmarking tests, where ✓ [resp. —] indicates the presence [resp. absence] of said feature, to the best of our knowledge. Average F1 [resp. SPS] scores for the pairwise [resp. multiple] global alignment are given, computed as explained in the text. F1 score is defined as the harmonic mean of the sensitivity (Sen) and positive predictive value (PPV). SPS is defined as the number of correctly nucleotide pairs in the alignment produced by a given algorithm, divided by the total number of aligned nucleotide pairs in the reference alignment.

In this paper we provide a very fast, comprehensive software package capable of pairwise/multiple local/global/semiglobal alignment with *p*-values and *E*-values for statistical significance. Moreover, due to its speed and relatively good accuracy, the software can be used for whole-genome scans for homologues of an RNA as query, a similar modality as in the software RSEARCH [30]. This type of whole-genome scan is in contrast to Infernal [31], which requires a multiple alignment of distinct RNAs to construct a covariance model for whole-genome searches. Searching for homologues from only a single sequence is potentially useful in the case of *orphan* RNAs, very recently discovered RNAs that do not belong to any known RNA family.

## Materials and methods

Quadratic time alignment using affine gap cost is implemented in RNAmountAlign using the Gotoh method [32] with the following pseudocode, shown for the case of semiglobal alignment. In our query scan form of semiglobal alignment, if **a** = *a*_1_, …, *a_n_* is the query sequence and **b** = *b*_1_, …, *b_m_* is the current genomic window being searched for an occurrence of **a**, then there is no penalty for gaps occurring to the left of the nucleotide of **b** aligned with *a*_1_ nor for gaps occurring to the right of the nucleotide of **b** aligned with *a*_*m*_, although internal gaps are penalized. This is obtained by the following pseudocode, as also found in [33]. Let *g*(*k*) denote an affine cost for size *k* gap, defined by *g*(0) = 0 and *g*(*k*) = *g*_*i*_ + (*k* − 1) ⋅ *g*_*e*_ for positive gap initiation [resp. extension] costs *g*_*i*_ [resp. *g*_*e*_]. For query **a** = *a*_1_, …, *a_n_* and target **b** = *b*_1_, …, *b_m_*, define (*n* + 1) × (*m* + 1) matrices *M*, *P*, *Q* as follows: *M*_*i*,0_ = *g*(*i*) for all 1 ≤ *i* ≤ *n*, *M*_0,*j*_ = 0 for all 1 ≤ *j* ≤ *m*, while for positive *i*, *j* we have *M*_*i*,*j*_ = max (*M*_*i*−1,*j*−1_ + *sim* (*a*_*i*_, *b*_*j*_), *P*_*i*,*j*_, *Q*_*i*,*j*_), where *sim* is a similarity measure formally defined later in Eq (17). For 1 ≤ *i* ≤ *n*, 1 ≤ *j* ≤ *m*, let *P*_0,*j*_ = 0 and *P*_*i*,*j*_ = max(*M*_*i*−1,*j*_ + *g*_*i*_, *P*_*i*−1,*j*_ + *g*_*e*_), and define *Q*_*i*,0_ = 0 and *Q*_*i*,*j*_ = max(*M*_*i*,*j*−1_ + *g*_*i*_, *Q*_*i*,*j*−1_ + *g*_*e*_, 0). Determine the maximum semiglobal alignment score in row *n*, then perform backtracking to obtain an optimal semiglobal alignment.

### Incremental ensemble mountain height

A secondary structure for a given RNA nucleotide sequence **a** = *a*_1_, …, *a_n_* is a set *s* of base pairs (*i*, *j*), where 1 ≤ *i* < *j* ≤ *n*, such that:

1. if (*i*, *j*) ∈ *s* then *a*_*i*_, *a*_*j*_ form either a Watson-Crick (AU,UA,CG,GC) or wobble (GU,UG) base pair,
2. if (*i*, *j*) ∈ *s* then *j* − *i* > *θ* = 3 (a steric constraint requiring that there be at least *θ* = 3 unpaired bases between any two positions that are paired),
3. if (*i*, *j*) ∈ *s* then for all *i*′ ≠ *i* and *j*′ ≠ *j*, (*i*′, *j*) ∉ *s* and (*i*, *j*′) ∉ *s* (nonexistence of base triples),
4. if (*i*, *j*) ∈ *s* and (*k*, *ℓ*) ∈ *s*, then it is not the case that *i* < *k* < *j* < *ℓ* (nonexistence of pseudoknots).

Secondary structures can be depicted in several equivalent manners, but in this paper, we use the dot-bracket notation in which matching left and right parenthesis positions indicate base-paired nucleotides. For instance, the EMBL accession code, sequence and secondary structure of a 53 nt type III hammerhead ribozyme from peach latent mosaic viroid (PLMVd), obtained from the Rfam database [34], is given as follows

> AJ550901.1/282-334

12345678901234567890123456789012345678901234567890123

GAAGAGUCGCGCUAAGCGCACUGAUGAGUCUUUGAGAUAAGACGAAACUCUUC

.((((((.((((…))))……‥((((……‥))))…)))))).

Positions 1 and 53 (for instance) are unpaired, as indicated by a dot, while positions 2 and 52 are paired and form the outermost base pair (2, 52), positions 12, 16 are paired and base pair (12, 16) constitutes one of the two apical (hairpin) loops, while the other apical (hairpin) loop is closed by the base pair (31, 40), etc.

Given an RNA sequence **a** = *a*_1_, …, *a_n_*, the base pairing probabilities $p_{i,j}^{a}$ are defined by

$$\begin{array}{cc} p_{i,j}^{a} & =\sum_{s\in S_{a},\left(i,j\right)\in s}\frac{\text{exp}(-E(s)/RT)}{Z}\\ Z & =\sum_{s\in S_{a}}\text{exp}\left(-E\left(s\right)/RT\right) \end{array}$$ (1)

where *Z* is the *partition function*, $S_{a}$ denotes the set of secondary structures of **a**, *E*(*s*) is the free energy of secondary structure *s* using energy parameters from [35]. Given **a** = *a*_1_, …, *a_n_* of length *n*, the *mountain height* [36] *h*_*s*_(*k*) of a secondary structure *s* of **a** at position *k* is defined as the number of base pairs in *s* that lie between an external loop and *k*, formally given by

$$\begin{array}{c} h_{s}\left(k\right)=|\{(i,j)\in s:i\leq k\}|-|\{(i,j)\in s:j\leq k\}| \end{array}$$ (2)

We follow [2, 36] in our definition of mountain height, and related notions of ensemble mountain height and distance, while [37] and Vienna RNA package [4] differ in an inessential manner by defining *h*_*s*_(*k*) = |{(*i*, *j*) ∈ *s*: *i* < *k*}| − |{(*i*, *j*) ∈ *s*: *j* ≤ *k*}|. If we consider the secondary structure *s*, defined as a *set* of base pairs (*i*, *j*) where 1 ≤ *i* < *j* ≤ *n*, as a dot-bracket notation, then *h*_*s*_(*k*) is simply the running count, where in scanning from left-to-right we add 1 to the count for each open parenthesis (, and subtract 1 from the count for each closed parenthesis ), encountered between 1, …, *k*.

The *ensemble mountain height* 〈*h*(*k*)〉 [37] for RNA sequence **a** = *a*_1_, …, *a_n_* at position *k* is defined as the average mountain height, where the average is taken over the Boltzmann ensemble of all low-energy structures *s* of sequence **a**. If base pairing probabilities *p*_*i*,*j*_ have been computed, then it follows that

$$\begin{array}{c} \left\langle h(k)\right\rangle =\sum_{i=1}^{k}\sum_{j=k+1}^{n}p_{i,j} \end{array}$$ (3)

and hence the *incremental ensemble mountain height*, which for values 1 < *k* ≤ *n* is defined by *m*_**a**_(*k*) = 〈*h*(*k*)〉 − 〈*h*(*k* − 1)〉 can be computed by

$$\begin{array}{c} m_{a}\left(k\right)=\sum_{j=k+1}^{n}p_{k,j}-\sum_{i=1}^{k}p_{i,k} \end{array}$$ (4)

**Fig 1:**
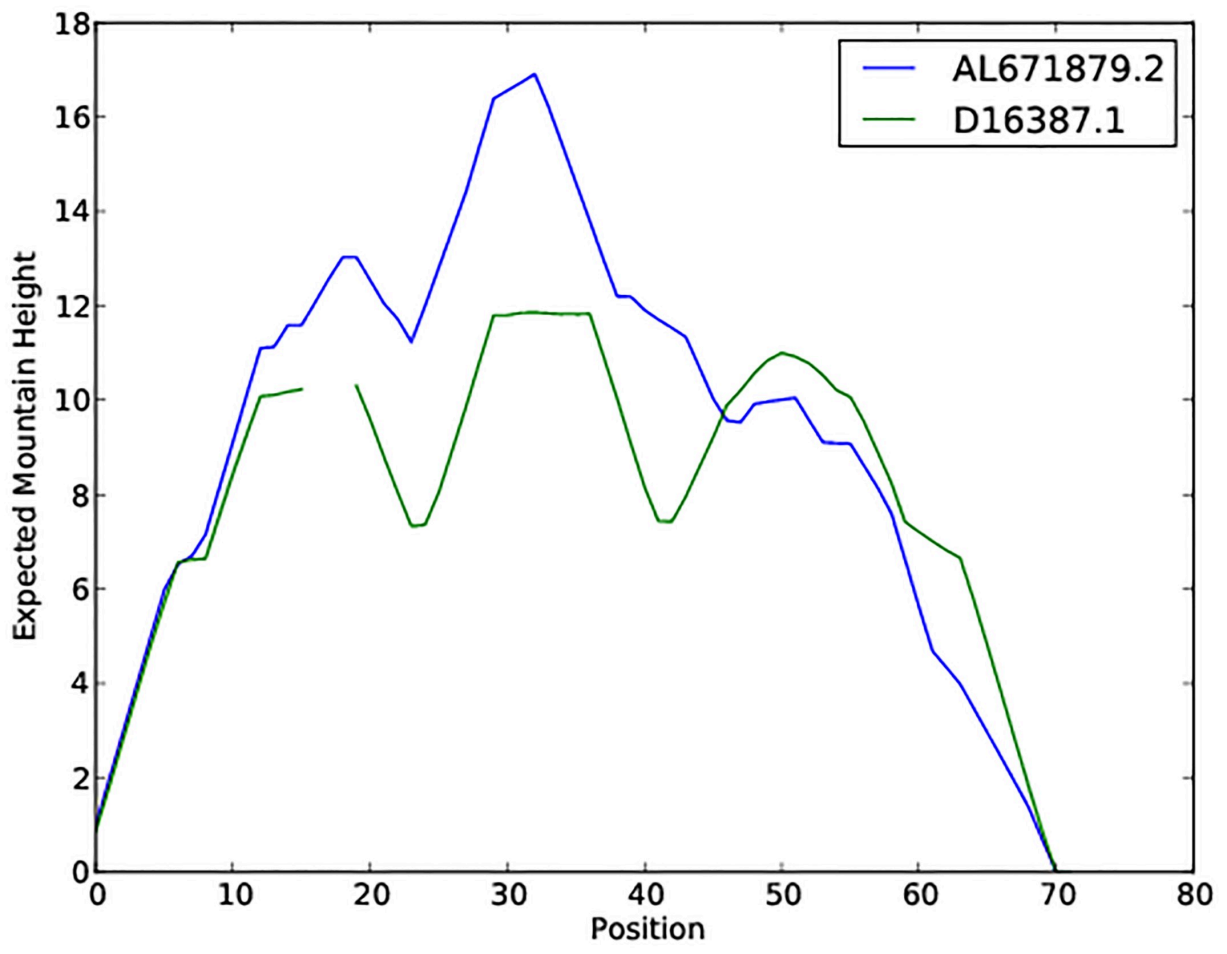
Ensemble mountain height display of the alignment computed by RNAmountAlign for 72 nt tRNA AL671879.2 and 69 nt tRNA D16387.1, both taken from BRAliBase 2.1 K2, using default gap parameters of −3 for gap initiation and −1 for gap extension, and structural weight *γ* = 1/2. The alignment produced by RNAmountAlign is identical to the reference alignment from BRAliBase 2.1 K2, although sequence identity is only 28% (twilight zone). The gap in the D16387.1 curve above corresponds to the size 3 gap in the alignment described in the text.

It is clear that −1 ≤ *m*_**a**_(*k*) ≤ 1, and that both ensemble mountain height and incremental ensemble mountain height can be computed in time that is quadratic in sequence length *n*, provided that base pairing probabilities *p*_*i*,*j*_ have been computed. Except for the cubic time taken by a C-library function call of export_bppm() from from Vienna RNA package [4], the software RNAmountAlign has quadratic time and space requirements. The following pairwise alignment of 72 nt tRNA AL671879.2 and 69 nt tRNA D16387.1, both taken from the BRAliBase 2.1 K2 database [38], was generated by the RNAmountAlign web server, with default parameters of gap initiation −3, gap extension −1 and structural weight *γ* = 1/2, which latter equally weights the contributions from sequence and structural similarity. The alignment produced by RNAmountAlign is identical to the reference alignment from BRAliBase 2.1 K2, although sequence identity is only 28% (twilight zone). The consensus structure, shown in S1 Fig, is produced by a call of RNAalifold from Vienna RNA Package, given the alignment produced by RNAmountAlign; Fig 1 is an alternative display of this alignment as a superimposition of the (gapped) ensemble mountain heights of tRNA AL671879.2 and tRNA D16387.1.

> AL671879.2

GGGGAUGUAGCUCAGUGGUAGAGCGCAUGCUUCGCAUGUAUGAGGCCCCGGGUUCGAUCCCCGGCAUCUCCA

> D16387.1

GUUUCAUGAGUAUAGC---AGUACAUUCGGCUUCCAACCGAAAGGUUUUUGUAAACAACCAAAAAUGAAAUA

> RNAalifold consensus structure

(((((((‥((((…….)))).(((((…….)))))….((((((……))))))))))))).

### Transforming distance into similarity

In [39], Seller’s (distance-based) global pairwise alignment algorithm [40] was rigorously shown to be equivalent to Needleman and Wunsch’s (similarity-based) global pairwise alignment algorithm [8]. Recalling that Seller’s alignment distance is defined as the minimum, taken over all alignments of the sum of distances *d*(*x*, *y*) between aligned nucleotides *x*, *y* plus the sum of (positive) weights *w*(*k*) for size *k* gaps, while Needleman-Wunsch alignment similarity is defined as the maximum, taken over all alignments of the sum of similarities *s*(*x*, *y*) between aligned nucleotides *x*, *y* plus the sum of (negative) gap weights *g*(*k*) for size *k* gaps, Smith and Waterman [39] show that by defining

$$\begin{array}{c} d(x,y)=\underset{a,b\in \{A,C,G,U\}}{\text{max}}s\left(a,b\right)-s\left(x,y\right) \end{array}$$ (5)

$$\begin{array}{c} w(k)=\frac{k}{2}\cdot \underset{a,b\in \{A,C,G,U\}}{\text{max}}s\left(a,b\right)-g\left(k\right) \end{array}$$ (6)

and by taking the minimum distance, rather than maximum similarity, the Needleman-Wunsch algorithm is transformed into Seller’s algorithm. Though formulated here for RNA nucleotides, equivalence holds over arbitrary alphabets and similarity measures (e.g. BLOSUM62).

To improve the intuitive understanding of our structural distance measure *STRSIM*, we initially define a simple distance measure *d*_0_ between dot-bracket symbols. For dot-bracket symbol *x* ∈ { (, •, ) }, define the sign function by

$$\begin{array}{cc} \text{sign}(k) & =\{\begin{array}{cc} 1 & \text{if}\;x=\;\text{(}\;\\ 0 & \text{if}\;x=•\\ -1 & \text{if}\;x=\;\text{)}\; \end{array} \end{array}$$ (7)

Now define the distance *d*_0_(*x*, *y*) = |*sign*(*x*) − *sign*(*y*)| between dot-bracket symbols *x*, *y* ∈ { (, •, ) } by

$$\begin{array}{cc} d_{0}\left(x,y\right) & =\{\begin{array}{cc} 0 & \text{if}\;x=y\\ 1 & \text{if}\;\text{[}x=•,y\in \{\;\text{(}\;,\;\text{)}\;\}\text{]}\;\text{or}\;\text{[}x\in \{\;\text{(}\;,\;\text{)}\;\},y=•\text{]}\\ 2 & \text{if}\;\text{[}x=\;\text{(}\;,y=\;\text{)}\;\text{]}\;\text{or}\;\text{[}x=\;\text{)}\;,y=\;\text{(}\;\text{]} \end{array} \end{array}$$ (8)

Let $A=(\begin{array}{c} s_{1}^{*}\cdots s_{N}^{*}\\ t_{1}^{*}\cdots t_{N}^{*} \end{array})$ denote a fixed alignment between two arbitrary secondary structures *s*, *t* of (possibly different) lengths *n*, *m*, where $s_{i}^{*},t_{i}^{*}\in \left\{\;\text{(}\;,•,\;\text{)}\;,-\right\}$, the dash − denotes the gap symbol, and where a gap is never aligned above another gap—this follows the notational convention for representation of alignments in [41]. We define the *structural alignment distance* for *A* by summing $d_{0}\left(s_{i}^{*},t_{i}^{*}\right)$ over those positions *i* where neither character $s_{i}^{*},t_{i}^{*}$ is a gap symbol, then adding *w*(*k*) for all size *k* gaps in *A*.

Using incremental ensemble mountain height from Eq (4), we can generalize structural alignment distance from the simple case of comparing two dot-bracket representations of secondary structures to the more representative case of comparing the low-energy Boltzmann ensemble of secondary structures for RNA sequence **a** to that of RNA sequence **b**. Given RNA sequences **a** = *a*_1_, …, *a_n_* and **b** = *b*_1_, …, *b_m_*, and given a fixed sequence alignment $\left(\begin{array}{c} a_{1}^{*}\cdots a_{N}^{*}\\ b_{1}^{*}\cdots b_{N}^{*} \end{array}\right)$ let $A=(\begin{array}{c} m_{a}{\left(1\right)}^{*}\cdots m_{a}{\left(N\right)}^{*}\\ m_{b}{\left(1\right)}^{*}\cdots m_{b}{\left(N\right)}^{*} \end{array})$ denote the corresponding alignment between the incremental ensemble mountain heights *m*_**a**_(1) ⋯ *m*_**a**_(*n*) of **a** and and the incremental ensemble mountain heights *m*_**b**_(1) ⋯ *m*_**b**_(*m*) of **b**. Generalize structural distance *d*_0_ defined in Eq (8) to *d*_1_ defined by *d*_1_(*a*_*i*_, *b*_*j*_) = |*m*_*a*_(*i*) − *m*_*b*_(*j*)|, where *m*_*a*_(*i*) and *m*_*b*_(*j*) are real numbers in the interval [−1, 1], defined by Eq 4. Define *ensemble structural alignment distance* for *A* by summing *d*_1_(*a*_*i*_, *b*_*j*_) over all aligned positions *i*, *j* for which neither character is a gap symbol, then adding positive weight *w*(*k*) for all size *k* gaps. By Eqs (5) and (6), it follows that an equivalent *ensemble structural similarity* measure between two positions *a*_*i*_, *b*_*j*_, denoted *STRSIM*(*a*_*i*_, *b*_*j*_), is obtained by multiplying *d*_1_ and *w*(*k*) by −1:

$$\begin{array}{c} STRSIM\left(a_{i},b_{j}\right)=-\left|m_{a}\left(i\right)-m_{b}\left(j\right)\right| \end{array}$$ (9)

This equation will be used later, since our algorithm RNAmountAlign combines both sequence and ensemble structural similarity. Indeed, −|*m*_*a*_(*i*) − *m*_*b*_(*j*)| ∈ [−2, 0] with maximum value of 0 while RIBOSUM85-60, shown in Table 3, has similarity values in the interval [−1.86, 2.22]. In order to combine sequence with structural similarity, both ranges should be rendered comparable as shown in the next section.

**Table 3:** RIBOSUM85-60 similarity scores.

	A	C	G	U
A	+2.22	-1.86	-1.46	-1.39
C	-1.86	+1.16	-2.48	-1.05
G	-1.46	-2.48	+1.03	-1.74
U	-1.39	-1.05	-1.74	+1.65

Initial portion of RIBOSUM85-60 similarity matrix for RNA nucleotides from [30]. RIBOSUM matrices also contain base pairing substitution scores, currently unused by RNAmountAlign.

### Pairwise alignment

**Fig 2:**
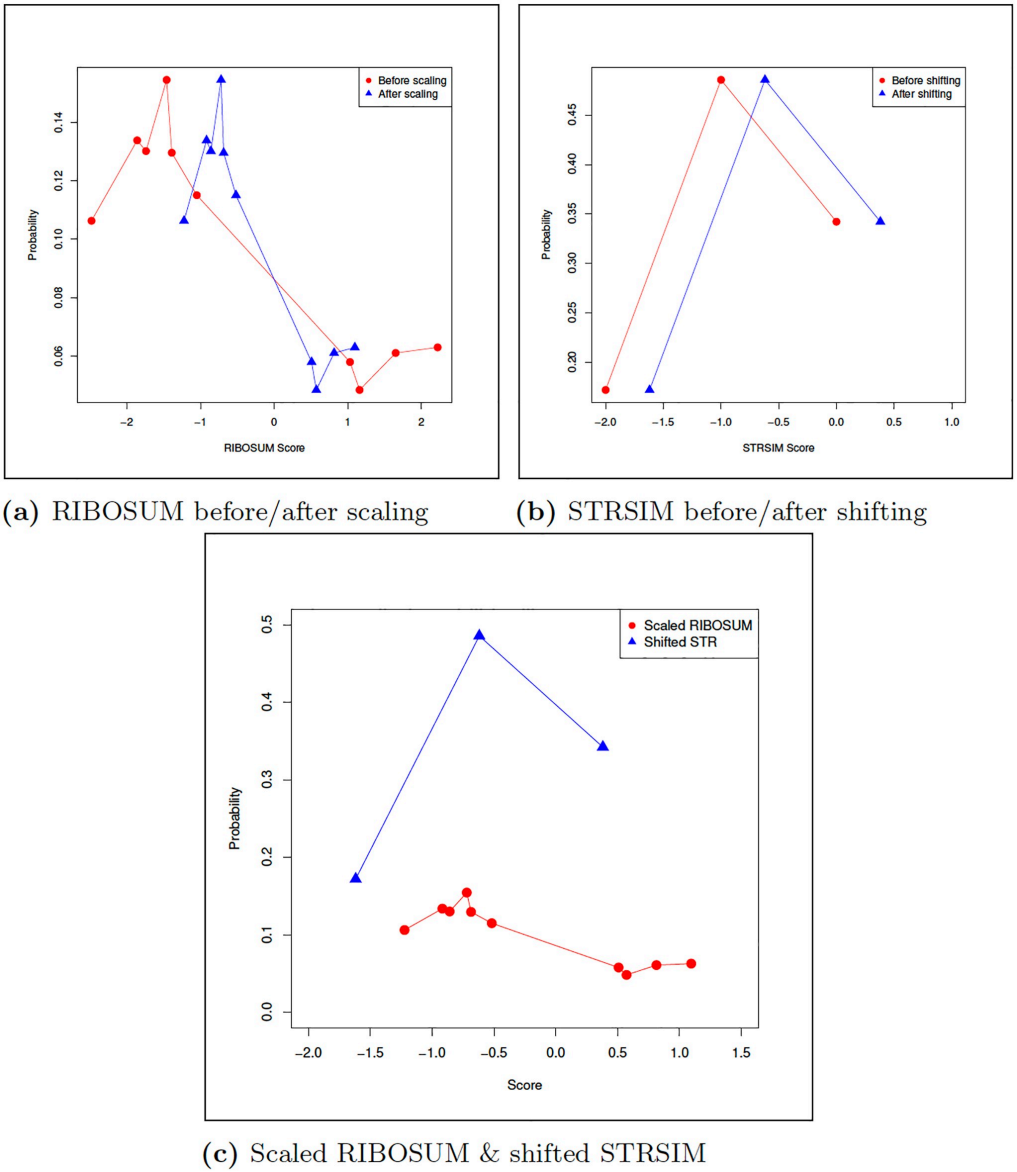
For 72 nt tRNA query sequence AL671879.2, there are 12 A’s, 20 C’s, 24 G’s, and 16 U’s so nucleotide relative frequencies are approximately *p*_*A*_ ≈ 0.167, *p*_*C*_ ≈ 0.278, *p*_*G*_ ≈ 0.333, *p*_*U*_ ≈ 0.222, and for 69 nt tRNA target sequence D16498.1, there are 26 A’s, 12 C’s, 12 G’s, and 19 U’s so nucleotide relative frequencies are approximately *p*_*A*_ ≈ 0.377, *p*_*C*_ ≈ 0.174, *p*_*G*_ ≈ 0.174, *p*_*U*_ ≈ 0.275. From the base pairing probabilities computed by RNAfold -p, we have query frequencies *p*_(_ = 0.3035, *p*_•_ = 0.3930, *p*_)_ = 0.3035 and target frequencies *p*_(_ = 0.2835, *p*_•_ = 0.433, *p*_)_ = 0.2835, so by Eqs (10), (11), (13) and (14), we have *μ*_seq_ = −0.9098, *σ*_seq_ = 1.5871 and *μ*_str_ = −0.8298, *σ*_str_ = 0.6967. By Eqs (15) and (16), we determine that RIBOSUM scaling factor *α*_seq_ = 0.4390 and *α*_str_ = 0.4305 (all values shown rounded to 4-decimal places). Using these values, the scaled RIBOSUM mean is now −0.39936, now equal to the shifted STRSIM mean, and both the scaled RIBOSUM standard deviation and shifted STRSIM standard deviation equal 0.6967. Panels (a) resp. (b) show the distribution of RIBOSUM resp. STRSIM values for the nucleotide and base pairing probabilities determined from query and target, while panel (c) shows the distribution of *α*_seq_-scaled RIBOSUM and *α*_str_-shifted STRSIM values. It follows that the distributions in panel (c) have the same (negative) mean and same standard deviation.

In order to combine sequence and ensemble structural similarity, we determine a multiplicative scaling factor *α*_seq_ and an additive shift factor *α*_str_ such that the mean and standard deviation for the distribution of sequence similarity values from a RIBOSUM matrix [30] (after being multiplied by *α*_seq_) are equal to the mean and standard deviation for the distribution of structural similarity values from STRSIM (after additive shift of *α*_str_). The RIBOSUM85-60 nucleotide similarity matrix used in this paper is given in Table 3, and the distributions for RIBOSUM and STRSIM values are shown in Fig 2 for the 72 nt transfer RNA AL671879.2. Given query **a** = *a*_1_, …, *a_n_* [resp. target **b** = *b*_1_, …, *b_n_*], let *p*_*A*_, *p*_*C*_, *p*_*G*_, *p*_*U*_ [resp. $p_{A}^{\prime },p_{C}^{\prime },p_{G}^{\prime },p_{U}^{\prime }$] denote the nucleotide relative frequencies for **a** [resp. **b**], i.e. the proportion of occurrences of each nucleotide A,C,G,U in query **a** [resp. target **b**]. Define the mean *μ*_seq_ and standard deviation *σ*_seq_ of RIBOSUM nucleotide similarities by

$$\begin{array}{c} \mu_{\text{seq}}=\sum_{x,y\in \{A,C,G,U\}}p_{x}p_{y}^{\prime }\cdot RIBOSUM\left(x,y\right) \end{array}$$ (10)

$$\begin{array}{c} \sigma_{\text{seq}}=\sqrt{\sum_{x,y\in \{A,C,G,U\}}p_{x}p_{y}^{\prime }\cdot RIBOSUM{\left(x,y\right)}^{2}-\mu_{\text{seq}}^{2}} \end{array}$$ (11)

Compute the base pairing probabilities $p_{i,j}^{a}$ of query sequence **a** for 1 ≤ *i* ≤ *j* ≤ *n*, and $p_{i,j}^{b}$ of target sequence **b** for 1 ≤ *i* ≤ *j* ≤ *m* by a call to the matrix bppm, using the Vienna RNA Package C-library.

Define the *expected* left $p_{\;\text{(}\;}^{a}$ and right $p_{\;\text{)}\;}^{a}$ base pairing probabilities, and the *expected* unpaired probability $p_{•}^{a}$ of query sequence **a** by the following

$$\begin{array}{cc} p_{\;\text{(}\;}^{a} & =\frac{1}{n}\cdot \sum_{k=1}^{n}\sum_{j=k+1}^{n}p_{k,j}^{a}\\ p_{\;\text{)}\;}^{a} & =\frac{1}{n}\cdot \sum_{k=1}^{n}\sum_{i=1}^{k-1}p_{i,k}^{a}\\ p_{•}^{a} & =1-p_{\;\text{(}\;}^{a}-p_{\;\text{)}\;}^{a} \end{array}$$ (12)

Analogously define the corresponding probabilities $p_{\;\text{(}\;}^{b}$, $p_{\;\text{)}\;}^{b}$, $p_{•}^{b}$ for the target sequence **b**.

Setting *s*_0_(*x*, *y*) = −*d*_0_(*x*, *y*), where *d*_0_(*x*, *y*) is defined in Eq (8), for given query [resp. target] base pairing probabilities *p*_(_, *p*_•_, *p*_)_ [resp. $p_{\;\text{(}\;}^{\prime },p_{•}^{\prime },p_{\;\text{)}\;}^{\prime }$] of dot-bracket characters, it follows that the mean *μ*_str_ and standard deviation *σ*_str_ of structural similarities can be computed by

$$\begin{array}{c} \mu_{\text{str}}=\sum_{x,y\in \{\;\text{(}\;,•,\;\text{)}\;\}}p_{x}p_{y}^{\prime }\cdot s_{0}\left(x,y\right) \end{array}$$ (13)

$$\begin{array}{c} \sigma_{\text{str}}=\sqrt{\sum_{x,y\in \{\;\text{(}\;,•,\;\text{)}\;\}}p_{x}p_{y}^{\prime }\cdot s_{0}{\left(x,y\right)}^{2}-\mu_{\text{str}}^{2}} \end{array}$$ (14)

Now we compute a multiplicative factor *α*_seq_ and an additive shift term *α*_str_, both dependent on frequencies *p*_*A*_, *p*_*C*_, *p*_*G*_, *p*_*U*_ and *p*_(_, *p*_•_, *p*_)_, such that the mean [resp. standard deviation] of nucleotide similarity multiplied by *α*_seq_ is equal to the mean [resp. standard deviation] of structural similarity after addition of shift term *α*_str_:

$$\begin{array}{cc} \alpha_{\text{seq}} & =\sigma_{\text{str}}/\sigma_{\text{seq}} \end{array}$$ (15)

$$\begin{array}{c} \alpha_{\text{str}}=\alpha_{\text{seq}}\cdot \mu_{\text{seq}}-\mu_{\text{str}} \end{array}$$ (16)

Given the query RNA **a** = *a*_1_, …, *a_n_* and target RNA **b** = *b*_1_, …, *b_m_* with incremental ensemble mountain heights *m*_**a**_(1) ⋯ *m*_**a**_(*m*) of **a**, *m*_**b**_(1) ⋯ *m*_**b**_(*m*) of **b**, and user-defined weight 0 ≤ *γ* ≤ 1, our final similarity measure is defined by

$$\begin{array}{cc} {\text{sim}}_{\gamma }\left(a_{i},b_{j}\right) & =\left(1-\gamma \right)\cdot \alpha_{\text{seq}}\cdot RIBOSUM\left(a_{i},b_{j}\right)\\ & +\gamma \cdot (\alpha_{\text{str}}+STRSIM\left(a_{i},b_{j}\right)) \end{array}$$ (17)

where *α*_seq_, *α*_str_ are computed by Eqs (15) and (16) depending on probabilities *p*_*A*_, *p*_*C*_, *p*_*G*_, *p*_*U*_ [resp. $p_{A}^{\prime },p_{C}^{\prime },p_{G}^{\prime },p_{U}^{\prime }$] and *p*_(_, *p*_•_, *p*_)_ [resp. $p_{\;\text{(}\;}^{\prime },p_{•}^{\prime },p_{\;\text{)}\;}^{\prime }$] of the query [resp. target]. All benchmarking computations were carried out using *γ* = 1/2, although it is possible to use position-specific weight *γ*_*i*,*j*_ defined as the average probability that *i* is paired in **a** and *j* is paired in **b**.

Our structural similarity measure is closely related to that of STRAL, which we discovered only after completing a preliminary version of this paper. Let $pl_{i}^{a}=\sum_{j<i}p_{j,i}^{a}$ and $pr_{i}^{a}=\sum_{j>i}p_{i,j}^{a}$ be the probability that position *i* of sequence **a** is paired to a position on the left or right, respectively. The similarity measure used in STRAL is defined by

$$\begin{array}{cc} {\text{sim}}_{\gamma }^{STRAL}\left(a_{i},b_{j}\right) & =\gamma \cdot (\sqrt{pl_{i}^{a}\cdot pl_{j}^{b}}+\sqrt{pr_{i}^{a}\cdot pr_{j}^{b}})\\ & +\sqrt{\left(1-pr_{i}^{a}-pl_{i}^{a}\right)\cdot \left(1-pr_{i}^{a}-pl_{i}^{a}\right)}\cdot RIBOSUM\left(a_{i},b_{j}\right) \end{array}$$ (18)

From Eqs (17) and (4) our measure can be defined as

$$\begin{array}{cc} {\text{sim}}_{\gamma }\left(a_{i},b_{j}\right) & =\gamma \cdot (\alpha_{\text{str}}-\left|\left(pr_{i}^{a}-pl_{i}^{a}\right)-\left(pr_{j}^{b}-pl_{j}^{b}\right)\right|)\\ & +\left(1-\gamma \right)\cdot \alpha_{\text{seq}}\cdot RIBOSUM\left(a_{i},b_{j}\right) \end{array}$$ (19)

Though clearly very related, RNAmountAlign was developed independently, without knowledge of STRAL, and offers a number of functionalities unavailable in STRAL, which latter appears to be no longer maintained. For instance, RNAmountAlign supports local and semiglobal alignment, and reports *p*-values and E-values; these features are not available in STRAL. Since we were unable to compile STRAL, our benchmarking results for STRAL use an adaptation of our code to support Eq (18). There are nevertheless some differences in how progressive alignment is implemented in STRAL that could affect run time.

To illustrate the method, suppose that the query [resp. target] sequence is the 72 nt tRNA AL671879.2 [resp. 69 nt tRNA D16498.1] with sequence GGGGAUGUAG CUCAGUGGUA GAGCGCAUGC UUCGCAUGUA UGAGGCCCCG GGUUCGAUCC CCGGCAUCUC CA. Then nucleotide query [resp. target] probabilities are (approximately) *p*_*A*_ ≈ 0.167, *p*_*C*_ ≈ 0.278, *p*_*G*_ ≈ 0.333, *p*_*U*_ ≈ 0.222. For the 69 nt tRNA target sequence D16498.1 with sequence GUUUCAUGAG UAUAGCAGUA CAUUCGGCUU CCAACCGAAA GGUUUUUGUA AACAACCAAA AAUGAAAUA the nucleotide relative frequencies are approximately $p_{A}^{\prime }\approx 0.377$, $p_{C}^{\prime }\approx 0.174$, $p_{G}^{\prime }\approx 0.174$, $p_{U}^{\prime }\approx 0.275$. From the base pairing probabilities returned by RNAfold -p [4], we determine that *p*_(_ = 0.3035, *p*_•_ = 0.3930, *p*_)_ = 0.3035 [resp. $p_{\;\text{(}\;}^{\prime }=0.2835$, $p_{•}^{\prime }=0.433$, $p_{\;\text{)}\;}^{\prime }=0.2835$]. Using these probabilities in Eqs (10)–(14), we determine that *μ*_seq_ = −0.9098, *σ*_seq_ = 1.5871 and *μ*_str_ = −0.8298, *σ*_str_ = 0.6967. By Eqs (15) and (16), we determine that RIBOSUM scaling factor *α*_seq_ = 0.4390 and *α*_str_ = 0.4305 (all values shown rounded to 4-decimal places). Using these values, the scaled RIBOSUM mean is now −0.39936, now equal to the shifted STRSIM mean, and both the scaled RIBOSUM standard deviation and shifted STRSIM standard deviation equal 0.6967. Since the mean and standard deviation of *α*_seq_-scaled RIBOSUM values are identical with that of *α*_str_-shifted STRSIM values, hence can be combined in Eq (17). Although sequence identity of the reference alignment of these tRNAs from BRAliBase 2.1 K2 is only 28%, the global alignment produced by RNAmountAlign is identical to that the reference alignment, using default parameters of gap initiation −3, gap extension −1, and structural weight *γ* = 1/2 in Eq (17).

Fig 2 depicts the distribution of RIBOSUM85-60 [resp. STRSIM] values in this case, both before and after application of scaling factor *α*_seq_ [resp. shift *α*_str_]—recall that *α*_seq_ and *α*_str_] depend on *p_A_*, *p_C_*, *p_G_*, *p_U_*, *p*_(_, *p*_•_, *p*_)_ of tRNA AL671879.2 and $p_{A}^{\prime },p_{C}^{\prime },p_{G}^{\prime },p_{U}^{\prime },p_{\;\text{(}\;}^{\prime },p_{•}^{\prime },p_{\;\text{)}\;}^{\prime }$ of tRNA D16498.1.

### Statistics for pairwise alignment

#### Karlin-Altschul statistics for local pairwise alignment

For a finite alphabet *A* and similarity measure *s*, suppose that the expected similarity $\sum_{x,y\in A}p_{x}p_{y}\cdot s\left(x,y\right)$ is negative and that *s*(*x*, *y*) is positive for at least one choice of *x*, *y*. In the case of BLAST, amino acid and nucleotide similarity scores are integers, for which the Karlin-Altschul algorithm was developed [12]. In contrast, RNAmountAlign similarity scores are not integers (or more generally values in a lattice), because Eq (17) combines real-valued *α*_seq_-scaled RIBOSUM nucleotide similarities with real-valued *α*_str_-shifted STRSIM structural similarities, which depend on query [resp. target] probabilities *p_A_*, *p_C_*, *p_G_*, *p_U_*, *p*_(_, *p*_•_, *p*_)_ [resp. $p_{A}^{\prime },p_{C}^{\prime },p_{G}^{\prime },p_{U}^{\prime },p_{\;\text{(}\;}^{\prime },p_{•}^{\prime },p_{\;\text{)}\;}^{\prime }$]. For that reason, we use Theorem 1 of Karlin, Dembo and Kawabata [13], reformulated using the notation of this paper, where the similarity score *s*(*x*, *y*) for RNA nucleotides *x*, *y* is defined by Eq (17).

**Theorem 1 (Karlin, Dembo, Kawabata** [13]**)**

*Given similarity measure s between nucleotides in alphabet A* = {*A*, *C*, *G*, *U*}, *let* λ* *be the unique positive root of* $E\left[e^{s(x,y)}\right]=\sum_{x,y\in A}p_{x}p_{y}^{\prime }\cdot e^{\lambda s(x,y)}$, *and let random variable S*_*k*_ *denote the score of a length k gapless alignment*. *For large local alignment score z*,

$$\begin{array}{c} P(M>\frac{\text{ln}\;nm}{\lambda^{*}}+z)\leq \text{exp}(-K^{*}e^{-\lambda^{*}z}) \end{array}$$ (20)

*where M denotes maximal segment scores for local alignment of random RNA sequences a*_1_, …, *a*_*n*_ *and b*_1_, …, *b*_*m*_, *and where*

$$\begin{array}{c} K^{*}=\frac{\text{exp}(-2\sum_{k=1}^{\infty }\frac{1}{k}\cdot (E\left[e^{\lambda^{*}S_{k};S_{k}<0}\right]+P\left(S_{k}\geq 0\right))}{\lambda^{*}E\left[S_{k}e^{\lambda^{*}\cdot S_{k}}\right]} \end{array}$$ (21)

#### Fitting data to probability distributions

Data were fit to the normal distribution (ND) by the method of moments (i.e. mean and standard deviation were taken from data analysis). Data were fit to the extreme value distribution (EVD)

$$\begin{array}{c} P(x<s)=1-\text{exp}(-Ke^{\lambda s}) \end{array}$$ (22)

by an in-house implementation of maximum likelihood to determine λ, *K*, as described in supplementary information to [30]. Data were fit to the gamma distribution by using the function fitdistr(x, ‘gamma’) from the package MASS in the R programming language, which determines rate and shape parameters for the density function

$$\begin{array}{c} f(x,\alpha ,\lambda )=\frac{\lambda^{\alpha }x^{\alpha -1}e^{-\lambda x}}{\Gamma (\alpha )} \end{array}$$ (23)

with where *α* is the shape parameter, the rate is 1/λ, where λ is known as the scale parameter.

### Multiple alignment

Suppose *p*_*A*_, *p*_*C*_, *p*_*G*_, *p*_*U*_ are the nucleotide probabilities obtained after the concatenation of all sequences. Let *p*_(_, *p*_•_, *p*_)_ be computed by individually folding each sequence and taking the arithmetic average of probabilities of (, • and ) over all sequences. The mean and standard deviation of sequence and structure similarity are computed similar to Eqs (10)–(14).

$$\begin{array}{c} \mu_{\text{seq}}=\sum_{x,y\in \{A,C,G,U\}}p_{x}p_{y}\cdot RIBOSUM\left(x,y\right) \end{array}$$ (24)

$$\begin{array}{c} \sigma_{\text{seq}}=\sqrt{\sum_{x,y\in \{A,C,G,U\}}p_{x}p_{y}\cdot RIBOSUM{\left(x,y\right)}^{2}-\mu_{\text{seq}}^{2}} \end{array}$$ (25)

$$\begin{array}{c} \mu_{\text{str}}=\sum_{x,y\in \{\;\text{(}\;,•,\;\text{)}\;\}}p_{x}p_{y}\cdot s_{0}\left(x,y\right) \end{array}$$ (26)

$$\begin{array}{c} \sigma_{\text{str}}=\sqrt{\sum_{x,y\in \{\;\text{(}\;,•,\;\text{)}\;\}}p_{x}p_{y}\cdot s_{0}{\left(x,y\right)}^{2}-\mu_{\text{str}}^{2}} \end{array}$$ (27)

Sequence multiplicative scaling factor *α*_seq_ and the structure additive shift factor *α*_str_ are computed from these values using Eqs (15) and (16).

As in CLUSTAL [42] and the CLUSTAL Omega [43], our software RNAmountAlign implements progressive multiple alignment using the *Unweighted Pair Group Method with Arithmetic Mean* (UPGMA) [44] and p. 360 of [41]. In UPGMA, one first defines a similarity matrix *S*, where *S*[*i*, *j*] is equal to (maximum) pairwise sequence similarity of sequences *i* and *j*. A rooted tree is then constructed by progressively creating a parent node of the two closest siblings. Parent nodes are profiles (PSSMs) that represent alignments of two or more sequences, hence can be treated as pseudo-sequences in a straightforward adaptation of pairwise alignment to the alignment of profiles. Let’s consider an alignment of *N* sequences $A=(\begin{array}{c} a_{11}^{*}\cdots a_{1M}^{*}\\ \cdots \\ a_{N1}^{*}\cdots a_{NM}^{*} \end{array})$ composed of *M* columns. Let $A_{i}=\left\{a_{1i}^{*},a_{2i}^{*},\ldots ,a_{Ni}^{*}\right\}$ denote column *i* of the alignment (for 1 ≤ *i* ≤ *M*). Suppose *p*(*i*, *x*), for *x* ∈ {*A*, *C*, *G*, *U*, −}, indicates the probability of occurrence of a nucleotide or gap at column *i* of alignment *A*. Then sequence similarity SEQSIM between two columns is defined by

$$\begin{array}{c} SEQSIM\left(A_{i},A_{j}\right)=\sum_{x\in \{A,C,G,U,-\}}\sum_{y\in \{A,C,G,U,-\}}p\left(i,x\right)\cdot p\left(j,y\right)\cdot R\left(x,y\right) \end{array}$$ (28)

where

$$\begin{array}{c} R(x,y)=\{\begin{array}{cc} 0 & \text{if}\;x=-\;\text{or}\;y=-\\ RIBOSUM(x,y) & \text{otherwise} \end{array} \end{array}$$ (29)

The structural measure for a profile is computed from the incremental ensemble heights averaged over each column. Let *m*_*A*_(*i*) denote the arithmetic average of incremental ensemble mountain height at column *A*_*i*_

$$\begin{array}{c} m_{A}\left(i\right)=\frac{\sum_{1\leq j\leq N}m_{a_{j}^{*}}\left(i\right)}{N} \end{array}$$ (30)

where $m_{a_{j}^{*}}\left(i\right)$ is the incremental ensemble mountain height at position *i* of sequence $a_{j}^{*}$ obtained from Eq (4). Here, let $m_{a_{j}^{*}}\left(i\right)=0$ if $a_{ji}^{*}$ is a gap. Structural similarity between two columns is defined by

$$\begin{array}{c} STRSIM\left(A_{i},A_{j}\right)=-\left|m_{A}\left(i\right)-m_{A}\left(j\right)\right| \end{array}$$ (31)

Finally, the combined sequence/structure similarity is computed from

$$\begin{array}{cc} {\text{sim}}_{\gamma }\left(A_{i},A_{j}\right) & =\left(1-\gamma \right)\cdot \alpha_{\text{seq}}\cdot SEQSIM\left(A_{i},A_{j}\right)\\ & +\gamma \cdot (\alpha_{\text{str}}+STRSIM\left(A_{i},A_{j}\right)) \end{array}$$ (32)

## Benchmarking

### Accuracy measures

Sensitivity, positive predictive value, and F1 score for pairwise alignments were computed as follows. Let $A=(\begin{array}{c} a_{1}^{*}\cdots a_{N}^{*}\\ b_{1}^{*}\cdots b_{N}^{*} \end{array})$ denote an alignment, where *a*_*i*_, *b*_*i*_ ∈ {*A*, *C*, *G*, *U*, −}, and the aligned sequences include may contain occurrences of the gap symbol ‘−’, provided that not both $a_{i}^{*}$ and $b_{i}^{*}$ are gap symbols. The number TP of true positives [resp. FP of false positives] is the number of alignment pairs $\left(a_{i}^{*},b_{i}^{*}\right)$ in the predicted alignment that belong to [resp. do not belong to] the reference alignment. The sensitivity (*Sen*) [resp. positive predictive value (*PPV*)] of a predicted alignment is TP divided by reference alignment length [resp. TP divided by predicted alignment length]. The *F*1 score is the harmonic mean of sensitivity and PPV, so $F1=\frac{2}{\left(1/Sen)+(1/PPV\right)}$. For the computation of *Sen*, *PPV*, and *F*1, we consider not only nucleotide pairs (*a*_*i*_, *b*_*j*_), but also include pairs of the form (*a_i_*, —) [resp. (—, *b_j_*)], which represent an insertion in the top sequence of alignment A (equivalently a deletion from the bottom sequence of A) [resp. deletion from the top sequence of A (equivalently an insertion in the bottom sequence of A)]. Suppose that the (toy) alignment A

$$\begin{array}{c} \begin{array}{ccccccc} & a_{1}^{*} & a_{2}^{*} & a_{3}^{*} & a_{4}^{*} & a_{5}^{*} & a_{6}^{*}\\ & a_{1} & a_{2} & a_{3} & - & a_{4} & a_{5}\\ a= & A & C & G & - & U & A\\ b= & A & - & - & C & U & A\\ & b_{1} & - & - & b_{2} & b_{3} & b_{4}\\ & b_{1}^{*} & b_{2}^{*} & b_{3}^{*} & b_{4}^{*} & b_{5}^{*} & b_{6}^{*} \end{array} \end{array}$$

is produced by an algorithm (such as RNAmountAlign, FOLDALIGN, MXSCARNA, etc.). Then **a** = *a*_1_*a*_2_*a*_3_*a*_4_*a*_5_, **a** = *ACGUA*, **b** = *b*_1_*b*_2_*b*_3_*b*_4_, **b** = *ACUA*. The length of alignment A is *N* = 6, while $a_{1}^{*}\cdots a_{6}^{*}=\text{ACG}-\text{UA}$ and $b_{1}^{*}\cdots b_{6}^{*}=A--\text{CUA}$. If B is the (toy) alignment of the same sequences

$$\begin{array}{c} \begin{array}{cccccc} & c_{1}^{*} & c_{2}^{*} & c_{3}^{*} & c_{4}^{*} & c_{5}^{*}\\ & a_{1} & a_{2} & a_{3} & a_{4} & a_{5}\\ a= & A & C & G & U & A\\ b= & A & C & - & U & A\\ & b_{1} & b_{2} & - & b_{3} & b_{4}\\ & d_{1}^{*} & d_{2}^{*} & d_{3}^{*} & d_{4}^{*} & d_{5}^{*} \end{array} \end{array}$$

taken from a reference database (such as BRAliBase 2.1), then sequences **a**, **b** are as above, the length of alignment B is *M* = 5, while $c_{1}^{*}\cdots c_{5}^{*}=\text{ACGUA}$ and $d_{1}^{*}\cdots d_{5}^{*}=\text{AC}-\text{UA}$. The number TP of correctly aligned pairs is 4, since

$$\begin{array}{c} \left(a_{1},b_{1}\right)=\left(a_{1}^{*},b_{1}^{*}\right)=\left(A,A\right)=\left(c_{1},d_{1}\right)=\left(c_{1}^{*},d_{1}^{*}\right),\\ \left(a_{4},b_{3}\right)=\left(a_{5}^{*},b_{5}^{*}\right)=\left(U,U\right)=\left(c_{4},d_{3}\right)=\left(c_{4}^{*},d_{4}^{*}\right),\\ \left(a_{5},b_{4}\right)=\left(a_{6}^{*},b_{6}^{*}\right)=\left(A,A\right)=\left(c_{5},d_{4}\right)=\left(c_{5}^{*},d_{5}^{*}\right),\\ \left(a_{3},-\right)=\left(a_{3}^{*},b_{3}^{*}\right)=\left(G,-\right)=\left(c_{3},-\right)=\left(c_{3}^{*},d_{3}^{*}\right), \end{array}$$

Because TP = 4, rather than 3 (if we had instead counted only aligned nucleotide pairs), the computations of *F*1 score, *Sen*, and *PPV* are duly affected.

To compare predicted structures with consensus Rfam structures, we computed the Matthews correlation coefficient (MCC) [45] as follows:

$$\begin{array}{c} MCC=\frac{TP_{str}\times TN_{str}-FP_{str}\times FN_{str}}{\sqrt{\left(TP_{str}+FP_{str}\right)\left(TP_{str}+FN_{str}\right)\left(TN_{str}+FP_{str}\right)+\left(TN_{str}+FN_{str}\right)}} \end{array}$$ (33)

where *TP*_*str*_ is the number of correctly predicted base pairs, *FP*_*str*_ is the number of incorrectly predicted base pairs, *TN*_*str*_ is the number of potential base pairs absent in both predicted and reference structures and *FN*_*str*_ is the number of base pairs in the reference structure that were not predicted.

In the case of local alignment, since the size of the reference alignment is unknown, only the predicted alignment length and PPV are reported. To compute the accuracy of multiple alignment, we used *sum-of-pair-scores* (SPS) [11], defined as the number of correctly nucleotide pairs in the alignment produced by a given algorithm, divided by the total number of aligned nucleotide pairs in the reference alignment. For completeness, and to contrast this with our definition of sensitivity, PPV and F1 score, we formally define SPS as follows. Suppose that *A* denotes a multiple alignment of the form $A=(\begin{array}{c} a_{1,1}^{*}\cdots a_{1,M}^{*}\\ \cdots \\ a_{N,1}^{*}\cdots a_{N,M}^{*} \end{array})$. For 1 ≤ *i*, *j* ≤ *M*, 1 ≤ *k* ≤ *N* define *p*_*i*,*j*,*k*_ = 1 if the nucleotide $a_{i,k}^{*}$ is aligned with the nucleotide $a_{j,k}^{*}$ in both the reference and predicted alignments, and *p*_*i*,*j*,*k*_ = 0 otherwise. Sum-of-pairs score SPS is then the sum, taken over all *i*, *j*, *k*, of the *p*_*i*,*j*,*k*_. Though SPS can be considered as the average sensitivity, taken over all sequence pairs in the alignment, this is not technically correct in our case, since our definition of sensitivity also counts pairs of the form (*X*, —) and (—, *X*), where *X* ∈ {*A*, *C*, *G*, *U*}, from the reference alignment.

To measure the conservation of secondary structures in alignments, structural conservation index (SCI) was computed using RNAalifold [46]. RNAalifold computes SCI as the ratio of the free energy of the alignment, computed by RNAalifold, with the average minimum free energy of individual structures in the alignment. SCI values close to 1 [resp. 0] indicate high [resp. low] structural conservation. All computations made with Vienna RNA Package used version 2.1.7 [4] using default Turner 2004 energy parameters [35]).

### Dataset for global and local alignment comparison

**Fig 3:**
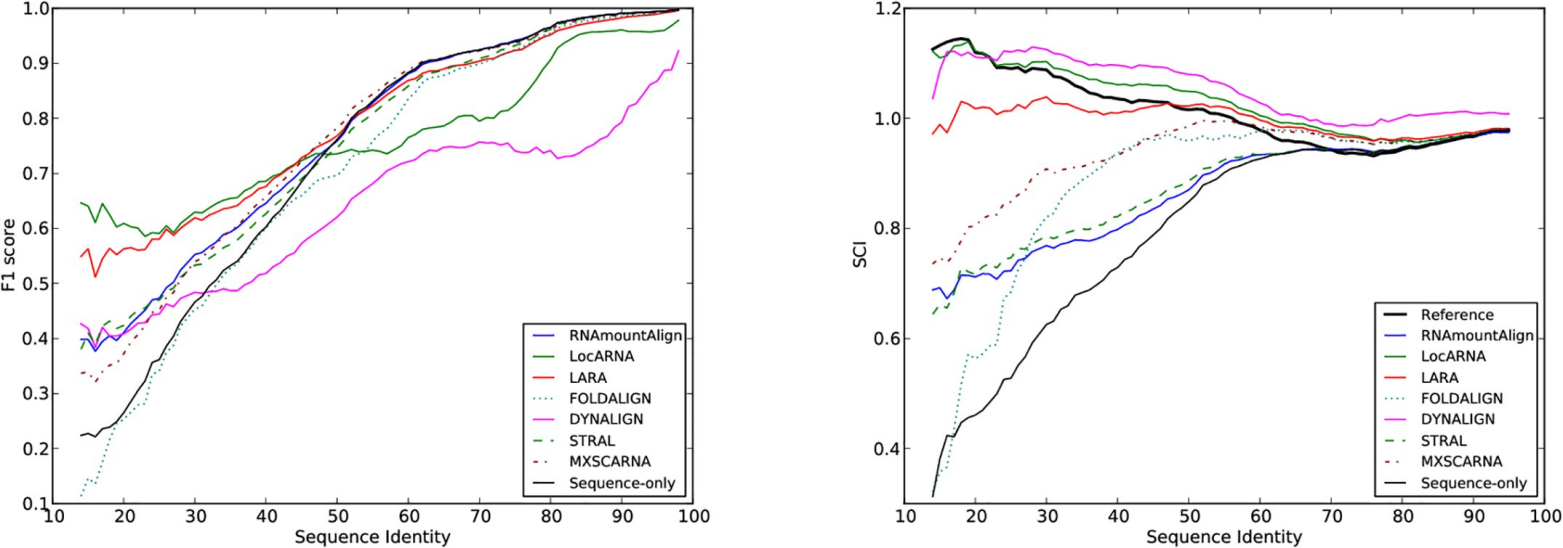
(Left) F1 score and (Right) structural conservation index (SCI) for *pairwise global alignments* using RNAmountAlign, LocARNA, LARA, FOLDALIGN, DYNALIGN, STRAL, MXSCARNA, and sequence-only(*γ* = 0). F1 score and SCI are shown as a function of reference alignment sequence identity for pairwise alignments in the BRAliBase 2.1 database used for benchmarking. Moving averages taken for centered, symmetric windows of size 11.

**Fig 4:**
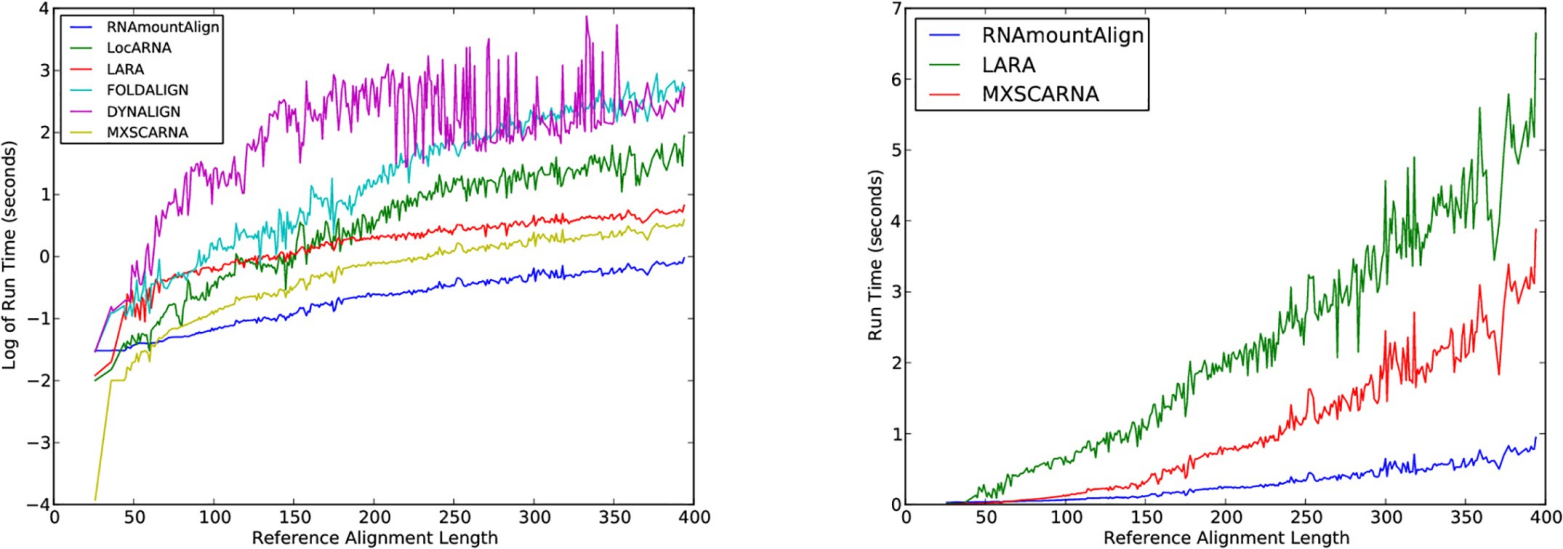
Run time of *pairwise global alignment* for RNAmountAlign, LocARNA, LARA, FOLDALIGN, DYNALIGN, and MXSCARNA. (Left) The log base 10 run time is shown as a function of reference alignment length for pairwise alignments in the BRAliBase 2.1 K2 database used for benchmarking. Moving averages taken for centered, symmetric windows of size 51. (Right) Actual run time for RNAmountAlign, LARA and MXSCARNA on the same data. Unlike the left panel the actual run time is shown, rather than log run time, without any moving average taken.

For *pairwise global* alignment benchmarking in Table 4, Figs 3 and 4, and S2–S4 Figs all 8, 976 pairwise alignments in k2 from BRAliBase 2.1 database [38] were used. Note that BRAliBase 2.1 does not include consensus secondary structures for the reference alignments, which are required in the computation of MCC. However, since BRAliBase 2.1 alignments are obtained from Rfam 7.0, we searched for the exact occurrences BRAliBase 2.1 sequences in Rfam 7.0 and computed their Rfam consensus structure. Some BRAliBase alignments are manually curated and thus different from Rfam alignments for which consensus structure could not be obtained. This produced 7, 154 pairwise BRAliBase alignments with their consensus structures. For the computation of MCC in S5 Fig, this subset of reference alignments were used.

**Table 4:** F1 scores for pairwise global alignment.

Type	NumAln	SeqId	MA(F)	LocARNA(F)	LARA(F)	FA(F)	DA(F)	STRAL(F)	MXSCARNA(F)
5.8S rRNA	76	0.72 ± 0.13	0.90 ± 0.09	0.82 ± 0.07	0.87 ± 0.15	0.89 ± 0.11	0.66 ± 0.22	0.88 ± 0.12	0.91 ± 0.09
5S rRNA	1162	0.60 ± 0.14	0.84 ± 0.16	0.87 ± 0.13	0.85 ± 0.16	0.86 ± 0.14	0.69 ± 0.17	0.82 ± 0.20	0.85 ± 0.14
Cobalamin	188	0.43 ± 0.10	0.56 ± 0.16	0.38 ± 0.17	0.49 ± 0.20	0.43 ± 0.24	0.36 ± 0.19	0.54 ± 0.17	0.57 ± 0.14
Entero 5 CRE	48	0.88 ± 0.06	0.98 ± 0.04	0.99 ± 0.04	0.99 ± 0.05	0.99 ± 0.02	0.87 ± 0.13	0.97 ± 0.06	0.98 ± 0.04
Entero CRE	65	0.80 ± 0.07	1.00 ± 0.00	0.99 ± 0.03	0.96 ± 0.07	0.99 ± 0.04	0.76 ± 0.17	1.00 ± 0.03	0.99 ± 0.02
Entero OriR	49	0.84 ± 0.06	0.95 ± 0.07	0.92 ± 0.09	0.94 ± 0.08	0.94 ± 0.07	0.84 ± 0.15	0.95 ± 0.07	0.96 ± 0.04
gcvT	167	0.44 ± 0.13	0.61 ± 0.19	0.61 ± 0.24	0.57 ± 0.25	0.40 ± 0.33	0.44 ± 0.19	0.62 ± 0.20	0.62 ± 0.18
Hammerhead 1	53	0.71 ± 0.17	0.89 ± 0.13	0.90 ± 0.11	0.87 ± 0.16	0.83 ± 0.25	0.52 ± 0.27	0.88 ± 0.16	0.87 ± 0.14
Hammerhead 3	126	0.66 ± 0.21	0.86 ± 0.20	0.88 ± 0.21	0.88 ± 0.20	0.80 ± 0.31	0.71 ± 0.31	0.90 ± 0.16	0.89 ± 0.16
HCV SLIV	98	0.85 ± 0.05	0.99 ± 0.03	0.98 ± 0.04	0.98 ± 0.03	0.99 ± 0.03	0.81 ± 0.34	0.99 ± 0.03	0.98 ± 0.03
HCV SLVII	51	0.83 ± 0.09	0.97 ± 0.06	0.96 ± 0.06	0.93 ± 0.10	0.95 ± 0.07	0.71 ± 0.22	0.95 ± 0.07	0.96 ± 0.06
HepC CRE	45	0.86 ± 0.06	1.00 ± 0.00	1.00 ± 0.00	1.00 ± 0.00	1.00 ± 0.00	0.77 ± 0.29	1.00 ± 0.00	1.00 ± 0.00
Histone3	84	0.78 ± 0.09	1.00 ± 0.00	1.00 ± 0.00	1.00 ± 0.00	1.00 ± 0.00	1.00 ± 0.00	1.00 ± 0.00	1.00 ± 0.00
HIV FE	733	0.87 ± 0.04	1.00 ± 0.02	1.00 ± 0.02	0.98 ± 0.05	0.99 ± 0.05	0.64 ± 0.29	1.00 ± 0.02	1.00 ± 0.02
HIV GSL3	786	0.86 ± 0.04	0.99 ± 0.02	0.99 ± 0.02	0.98 ± 0.05	0.99 ± 0.02	0.80 ± 0.19	0.99 ± 0.02	0.99 ± 0.02
HIV PBS	188	0.92 ± 0.02	1.00 ± 0.01	1.00 ± 0.01	1.00 ± 0.02	0.99 ± 0.03	0.91 ± 0.11	1.00 ± 0.01	0.99 ± 0.02
Intron gpII	181	0.46 ± 0.13	0.64 ± 0.17	0.64 ± 0.17	0.63 ± 0.17	0.50 ± 0.28	0.49 ± 0.18	0.65 ± 0.15	0.65 ± 0.15
IRES HCV	764	0.65 ± 0.11	0.88 ± 0.16	0.45 ± 0.19	0.86 ± 0.17	0.68 ± 0.38	0.85 ± 0.08	0.88 ± 0.08	0.89 ± 0.11
IRES Picorna	181	0.84 ± 0.07	0.97 ± 0.03	0.61 ± 0.04	0.96 ± 0.04	0.95 ± 0.04	0.85 ± 0.11	0.96 ± 0.04	0.96 ± 0.04
K chan RES	124	0.74 ± 0.10	0.99 ± 0.02	0.98 ± 0.05	0.89 ± 0.19	0.95 ± 0.08	0.58 ± 0.26	0.95 ± 0.11	0.96 ± 0.05
Lysine	80	0.50 ± 0.13	0.72 ± 0.13	0.54 ± 0.15	0.71 ± 0.18	0.66 ± 0.16	0.50 ± 0.16	0.72 ± 0.15	0.71 ± 0.15
Retroviral psi	89	0.88 ± 0.03	0.93 ± 0.03	0.93 ± 0.03	0.93 ± 0.03	0.92 ± 0.04	0.74 ± 0.12	0.93 ± 0.04	0.93 ± 0.03
S box	91	0.60 ± 0.10	0.75 ± 0.13	0.76 ± 0.16	0.79 ± 0.14	0.67 ± 0.24	0.54 ± 0.16	0.77 ± 0.12	0.80 ± 0.10
SECIS	114	0.44 ± 0.16	0.59 ± 0.21	0.62 ± 0.21	0.57 ± 0.25	0.54 ± 0.25	0.39 ± 0.24	0.61 ± 0.20	0.61 ± 0.19
sno 14q I II	44	0.75 ± 0.10	0.92 ± 0.10	0.89 ± 0.16	0.85 ± 0.20	0.89 ± 0.19	0.58 ± 0.27	0.91 ± 0.13	0.92 ± 0.10
SRP bact	114	0.48 ± 0.16	0.65 ± 0.21	0.66 ± 0.21	0.63 ± 0.25	0.65 ± 0.21	0.51 ± 0.22	0.61 ± 0.25	0.66 ± 0.21
SRP euk arch	122	0.51 ± 0.20	0.62 ± 0.29	0.35 ± 0.17	0.64 ± 0.28	0.64 ± 0.26	0.50 ± 0.26	0.61 ± 0.29	0.64 ± 0.27
T-box	18	0.68 ± 0.15	0.77 ± 0.17	0.49 ± 0.17	0.68 ± 0.25	0.70 ± 0.17	0.59 ± 0.21	0.74 ± 0.15	0.74 ± 0.14
TAR	286	0.87 ± 0.04	0.99 ± 0.03	0.99 ± 0.02	0.99 ± 0.03	0.98 ± 0.04	0.83 ± 0.19	0.99 ± 0.04	0.99 ± 0.02
THI	321	0.45 ± 0.10	0.68 ± 0.16	0.66 ± 0.20	0.68 ± 0.18	0.50 ± 0.29	0.48 ± 0.18	0.65 ± 0.20	0.69 ± 0.15
tRNA	2039	0.43 ± 0.12	0.75 ± 0.21	0.85 ± 0.16	0.82 ± 0.19	0.76 ± 0.27	0.66 ± 0.23	0.72 ± 0.22	0.75 ± 0.20
U1	82	0.63 ± 0.17	0.79 ± 0.17	0.70 ± 0.13	0.79 ± 0.19	0.80 ± 0.14	0.67 ± 0.20	0.77 ± 0.17	0.80 ± 0.17
U2	112	0.64 ± 0.16	0.75 ± 0.17	0.63 ± 0.13	0.76 ± 0.19	0.73 ± 0.22	0.59 ± 0.19	0.75 ± 0.18	0.76 ± 0.17
U6	30	0.83 ± 0.06	0.93 ± 0.05	0.89 ± 0.09	0.90 ± 0.08	0.88 ± 0.10	0.72 ± 0.14	0.93 ± 0.06	0.92 ± 0.07
UnaL2	138	0.77 ± 0.08	0.93 ± 0.08	0.92 ± 0.09	0.89 ± 0.15	0.91 ± 0.10	0.65 ± 0.29	0.94 ± 0.08	0.92 ± 0.06
yybP-ykoY	127	0.39 ± 0.14	0.58 ± 0.20	0.54 ± 0.23	0.57 ± 0.25	0.40 ± 0.33	0.46 ± 0.22	0.56 ± 0.20	0.55 ± 0.21
Pooled Average	249.33	0.63	0.84	0.81	0.84	0.80	0.68	0.82	0.84

Average F1 scores (± one standard deviation) for *pairwise global alignment* of RNAmountAlign and four widely used RNA sequence/structure alignment algorithms on the benchmarking set of 8,976 pairwise alignments from the BRaliBase K2 database [38]. For each indicated Rfam family, the the number of alignments (NumAln), sequence identity (SeqId), and F1 scores for RNAmountAlign, LocARNA, LARA, FOLDALIGN, and DYNALIGN are listed, along with pooled averages over all 8,976 pairwise alignments. Parameters used in Eq (17) for RNAmountAlign were similarity matrix RIBOSUM85-60, structural similarity weight *γ* = 1/2, gap initiation *g*_*i*_ = −3, gap extension *g*_*e*_ = −1.

For each pairwise alignment, a consensus structure was determined from the Rfam family consensus structure by removing gaps, removing base pairs (*i*, *j*) which are noncanonical or for which *j* − *i* ≤ *θ* = 3. These derived consensus structures were used as the reference structures in the computation of MCC. To be explicit, consider the following toy example of multiple alignment in Stockholm format—for illustrative purposes, pretend that this is an Rfam seed alignment of a small RNA family (recall that additional requirements must be met concerning number of sequences and average nucleotide length for an Rfam family to be selected).

# STOCKHOLM 1.0

positions   123456789012345678

Eg1      GGACAAGCAAUGCUUGCC

Eg2      GG-CAAGCA-UGCUUGAC

Eg3      GGACAAGCAAUGCUUGCC

#=GC SS_cons ≪-⋘≪___⋙⋙>

#=GC RF    GGACAAGCAAUGCUUGCC

This multiple alignment would then produce the following consensus structures for the *pairwise* alignment of Eg1 with Eg2.

> Eg1

123456789012345678

GGACAAGCAAUGCUUGCC

((.(((((…)))))))

> Eg2

1234567890123456

GGCAAGCAUGCUUGAC

(.((((….)))).)

Note that the Rfam consensus indicates that positions 2 and 17 are paired in the multiple sequence alignment, hence the reference structure for Eg1 has the GC base pair (2, 17) as expected. However, the the corresponding positions in Eg2 are G,A. To avoid violating the requirement (1) in the definition of secondary structure, the reference structure for Eg2 has dots at positions 2 and 15. Note that the Rfam consensus indicates a hairpin loop at positions 8 and 12 of the multiple sequence alignment, so the consensus structure for Eg1 contains the base pair (8, 12). However, since Eg2 contains A-U, this hairpin would contain only two nucleotides A,U, which would violate condition (2) of the definition of secondary structure. For that reason, there is no base pair (7, 10) in the reference structure of Eg2.

For *multiple global* alignment benchmarking, we used k5 BRAliBase 2.1, which includes 2,405 reference alignments, each composed of 5 sequences. For *pairwise local alignment* benchmarking, 75 pairwise alignments having sequence identity ≤ 70% were randomly selected from each of 20 well-known families from the Rfam 12.0 database [34], many of which were considered in a previous study [47], yielding a total of 1500 alignments. Following [48], these alignments were trimmed on the left and right, so that both first and last aligned pairs of the alignment do not contain a gap symbol. For sequences **a** = *a*_1_, …, *a_n_* [resp. **b** = *b*_1_, …, *b_m_*] from each alignment, random sequences **a**′ [resp. **b**′] were generated with the same nucleotide frequencies, then a random position was chosen in **a**′ [resp. **b**′] in which to insert **a** [resp. **b**], thus resulting in a pair of sequences of lengths 4*n* and 4*m*. Finally, since sequence identity was at most 70%, the RIBOSUM70-25 similarity matrix was used in RNAmountAlign. Preparation of the benchmarking dataset for local alignment was analogous to the method used in *multiple* local alignment of [48].

### Dataset for correlation of *p*-values for different distribution fits

A pool of 2220 sequences from the Rfam 12.0 database [34] was created as follows. One sequence was selected from each Rfam family having average sequence length at most 200 nt, with the property that the base pair distance between its minimum free energy (MFE) structure and the Rfam consensus structure was a minimum. For example, the 102 nt sequence with EMBL accession code AAOX01000028.142464-42565 was selected from family RF00167 since its MFE structure was identical to its Rfam consensus structure, while the 178 nt sequence with EMBL accession code AE016827.11325416-1325239 was selected from RF00168 since the base pair distance between its MFE structure and its Rfam consensus structure was 11—the base pair distance between MFE and consensus structure for all other sequences from RF00168 was greater than or equal to 11. Subsequently, for each of 500 randomly selected *query* sequences from the pool of 2220 sequences, 1000 random *target* sequences of length 400 nt were generated to have the same expected nucleotide frequency as that of the query. For each query and random target, five semiglobal alignments were created using gap initiation costs of *g*_*i*_ ∈ {−1, −2, −3, −4, −5} with gap extension cost *g*_*e*_ equal to one-third the gap initiation cost. For each alignment score *x* for query and random target, the *p*-value was computed as 1 − *CDF*(*x*) for ND, EVD and GD, where *CDF*(*x*) is the cumulative density function evaluated at *x*. Additionally, a heuristic *p*-value was determined by calculating the proportion of alignment scores for given query that exceed *x*.

### Software version and hardware specs

For benchmarking, we used LocARNA (version 1.8.7), FOLDALIGN (version 2.5), FOLDALIGNM (version 1.0.1), LARA (version 1.3.2), DYNALIGN (from version 5.7 of RNAstructure), the sequence alignment algorithm MUSCLE (version 3.8.31), STRAL (in-house implementation due to unavailability of the software [22], and MXSCARNA (version 2.1). For genome query scan benchmarking, we used RSEARCH 1.1, FOLDALIGN (version 2.5), and RNAmountAlignScan. Recommended default parameters were used for each software, including RNAmountAlign from this paper. The commands used to run the software are given in S1, S2 and S3 Tables. Each software package was run on a cluster of identically configured Intel Xeon 2.66 GHz 4-core nodes with 16GB of memory, running CentOS Linux release 6.10.

In contrast to all other benchmarking work described in this paper, benchmarking tests for RNAmountAlignScan, RSEARCH, and FOLDALIGN in genome scanning mode, as described in Section *Query Scan*, were conducted on a 24-processor Intel Xeon CPU E5-2440 2.40 GHz system with 198GB memory.

## Results

We benchmarked RNAmountAlign’s performance for pairwise and multiple alignments on BRAliBase K2 and K5 datasets, respectively.

### Pairwise alignment

Fig 3, S2 and S3 Figs depict moving averages of *pairwise global alignment* sensitivity, positive predictive value (PPV) and F1-score for the software described in this paper, as well as for LocARNA, FOLDALIGN, LARA, DYNALIGN, STRAL, and MXSCARNA. For pairwise benchmarking, reference alignments of size 2, a.k.a. K2, were taken from the BRAliBase 2.1 database [38]. BRAliBase 2.1 K2 data are based on seed alignments of the Rfam 7.0 database, and consist of 8,976 alignments of RNA sequences from 36 Rfam families.

Moving averages (window size 11) of sensitivity, positive predictive value, and F1 score were computed as a function of sequence identity, where it should be noted that the number of pairwise alignments for different values of sequence identity can vary for the BRAliBase 2.1 data (e.g. there are only 35 pairwise alignments having sequence identity < 20%). In computing moving averages, each value represents the average over a symmetric window (*k* − 5, *k* + 5) of size 11 nt centered at the value from the *x*-axis. For 504 pairwise global alignments, FOLDALIGN produced “No global alignment was found. This can either be due to the pruning, or because no structural alignment exists” and for 29, DYNALIGN yielded no alignments. The only BRAliBase alignment that both tools failed was Hammerhead_1.apsi36.sci72.no1. In our benchmarking the sensitivity, PPV and F1 values for such failures of FOLDALIGN and DYNALIGN were all assigned the value of 0, which explains the weaker performance of these two methods in Fig 3 compared to [14], where such failures are simply excluded from the analysis. Moreover, we observed a larger number of failed alignments in FOLDALIGN 2.5 than in the previous work of [14]. Default parameters were used for all software. For our software RNAmountAlign, gap initiation cost was -3, gap extension -1, and sequence/structure weighting parameter *γ* was 0.5 (value obtained by optimizing on a small set of 300 random alignments from Rfam 12.0, not considered in training or testing set). The sequence-only alignment is computed from RNAmountAlign with the same gap penalties, but for *γ* = 0. While its accuracy is high, RNAmountAlign is faster by an order of magnitude than LocARNA, LARA, FOLDALIGN, and DYNALIGN—indeed, algorithmic time complexity of our method is *O*(*n*^3^) for two sequences of length *n*. Since STRAL could not be compiled on any of our systems, we implemented its algorithm by modifying RNAmountAlign and obtained results for STRAL’s default parameter settings. Therefore, the run time of STRAL is identical to RNAmountAlign but RNAmountAlign achieves higher F1 score, sensitivity and PPV. MXSCARNA and RNAmountAlign have similar average F1 scores. Indeed, a paired 2-tailed Wilcoxson signed-rank test of the difference between F1 scores, as computed by MXSCARNA and RNAmountAlign, for the 8,976 pairwise global alignments mentioned in Table 4. It follows that the (null) hypothesis *H*_0_ cannot be rejected, where *H*_0_ asserts that the difference is 0 between F1 scores of MXSCARNA and RNAmountAlign—see S4 Table. Note however that the software MXSCARNA has slower run time than RNAmountAlign. Both MXSCARNA and RNAmountAlign support global and local alignment; however, unlike MXSCARNA, RNAmountAlign also supports semiglobal alignment and reports *p*-values. The right panel of Fig 4 depicts actual run times of the fastest software, RNAmountAlign, with the next fastest software, MXSCARNA and LARA. Unlike the graph in the left panel, actual run times are shown, graphed as a function of sequence length, rather than logarithms of moving averages.

In addition, Table 4 displays average pairwise global alignment F1 scores for RNAmountAlign, LocARNA, LARA, FOLDALIGN, DYNALIGN, STRAL, and MXSCARNA when benchmarked on 36 families from the BRaliBase K2 database comprising altogether 8,976 RNA sequences with average length of 249.33. Averaging over all sequences, the F1 scores for the programs just mentioned were respectively 0.8370, 0.7808, 0.8406, 0.7977, 0.6822, 0.8247, 0.8402; i.e. F1 score 0.8406 of LARA and 0.8402 of MXSCARNA slightly exceeded the F1 score 0.8370 of RNAmountAlign and 0.8247 of STRAL, while other methods trailed by several percentage points. S4 Table indicates the statistical significance of difference between all 8,976 F1 scores. Morevoer, S6 and S7 Tables display values for global alignment sensitivity and positive predictive value, benchmarked on the same data for the same programs—these results are similar to the F1 scores in Tables 2 and 4.

Although there appears to be no universally accepted criterion for quality of local alignments, Table 5 shows pairwise local alignment comparisons for the above-mentioned methods supporting local alignment: RNAmountAlign, FOLDALIGN, and LocARNA. We had intended to include SCARNA_LM [48] in the benchmarking of multiple local alignment software; however, SCARNA_LM no longer appears to be maintained (The SCARNA_LM web server is no longer functional, and the authors did not respond to our request for the source code of SCARNA_LM). Since the reference alignments for the local benchmarking dataset are not known, and sensitivity depends upon the length of the reference alignment, we only report local alignment length and positive predictive value. Abbreviating RNAmountAlign by MA, FOLDALIGN by FA, and LocARNA by LOC, Table 5 shows average run time in seconds of MA (2.30 ± 2.12), FA (625.53 ± 2554.61), LOC (5317.96 ± 8585.19), average alignment length of reference alignments (118.67 ± 47.86), MA (50.35 ± 42.33), FA (114.86 ± 125.33), LOC (556.82 ± 227.00), and average PPV scores MA (0.53 ± 0.42), FA (0.64 ± 0.36), LOC (0.03 ± 0.04). S5 Table presents *p*-values for a 2-tailed paired Wilcoxon signed-rank test whether the difference in positive predictive values fo 1,500 pairwise local alignments.

**Table 5:** Pairwise local alignment benchmarking.

TYPE	SEED(LENGTH)	MA(LENGTH)	MA(PPV)	MA(TIME)	FA(LENGTH)	FA(PPV)	FA(TIME)	LOC(LENGTH)	LOC(PPV)	LOC(TIME)
5 8S rRNA	158.48±7.40	71.20±41.55	0.80±0.32	3.70±0.43	168.33±89.23	0.75±0.25	509.56±411.83	767.67±43.35	0.01±0.03	9571.39±6152.56
5S rRNA	120.87±2.09	34.79±25.44	0.45±0.46	1.90±0.13	133.81±84.46	0.65±0.34	331.86±488.57	584.00±23.69	0.02±0.04	3093.17±1934.60
Cobalamin	221.03±13.67	28.60±16.77	0.57±0.44	7.67±1.14	451.73±256.29	0.22±0.28	6830.15±9052.56	1028.20±59.27	0.02±0.02	25712.40±15252.51
Hammerhead 3	64.24±11.08	31.88±20.40	0.38±0.42	0.38±0.11	36.91±31.83	0.30±0.41	23.95±11.81	279.05±38.70	0.04±0.06	159.87±123.44
let-7	85.73±3.11	55.37±28.14	0.75±0.22	0.89±0.10	72.95±27.35	0.48±0.33	65.51±28.66	390.76±21.37	0.04±0.05	462.12±283.01
Lysin	193.91±13.07	68.71±42.73	0.30±0.33	6.27±0.80	163.76±104.21	0.57±0.30	554.25±730.12	918.41±48.19	0.03±0.04	18690.26±10232.32
mir-10	75.71±1.27	55.09±21.97	0.67±0.24	0.72±0.04	66.91±30.83	0.48±0.36	45.68±19.80	358.55±15.96	0.03±0.04	333.63±227.10
Purine	102.01±0.93	129.05±86.84	0.41±0.39	1.37±0.07	69.80±6.70	0.88±0.15	87.27±30.47	497.41±16.81	0.03±0.05	2395.40±1571.67
RFN element	147.23±13.62	44.11±24.91	0.94±0.11	2.83±0.56	114.59±98.77	0.80±0.24	619.68±1289.50	687.71±62.46	0.03±0.05	5893.83±3827.59
S-box leader	120.13±16.14	50.35±30.00	0.57±0.36	1.68±0.44	88.72±60.79	0.79±0.21	190.03±493.08	554.09±55.21	0.03±0.04	2399.58±1484.64
SECIS	68.55±2.88	25.76±21.34	0.05±0.19	0.53±0.05	54.25±53.42	0.16±0.28	51.07±65.81	318.53±16.40	0.02±0.03	279.38±187.58
SNORD113	79.69±6.10	40.03±23.27	0.33±0.42	0.75±0.07	47.63±30.40	0.62±0.40	44.32±18.12	373.69±21.77	0.02±0.02	641.43±421.62
SRP bact	96.20±9.99	30.81±14.92	0.69±0.41	0.99±0.30	105.08±82.04	0.66±0.32	225.15±336.93	423.55±74.67	0.02±0.04	726.66±659.87
THI element	117.20±11.95	33.03±14.43	0.51±0.45	1.62±0.30	84.45±85.58	0.75±0.31	253.89±352.01	535.40±43.83	0.02±0.02	2319.39±1468.99
tRNA	76.05±5.79	37.31±45.09	0.23±0.40	0.70±0.09	62.15±38.30	0.67±0.40	73.45±78.89	360.29±24.06	0.02±0.04	479.15±265.22
Tymo tRNA-like	86.25±1.35	41.27±21.96	0.50±0.39	0.79±0.05	78.97±33.70	0.76±0.21	84.70±55.19	409.13±14.22	0.04±0.05	684.12±411.97
U1	167.16±2.58	48.36±32.73	0.69±0.34	4.52±0.16	221.36±121.42	0.61±0.23	1755.35±1255.41	804.19±24.78	0.03±0.05	11142.21±6902.37
U4	163.25±24.55	50.64±27.53	0.42±0.41	3.72±1.30	91.75±41.17	0.79±0.20	263.51±140.53	742.17±84.30	0.02±0.03	9361.29±5839.12
UnaL2	54.25±0.66	48.80±25.71	0.70±0.40	0.36±0.01	36.11±3.30	0.99±0.04	23.05±8.38	263.79±8.94	0.03±0.06	171.59±104.10
ykoK	175.39±7.32	82.05±58.19	0.68±0.36	4.67±0.45	147.55±69.66	0.81±0.20	472.79±583.01	844.27±31.56	0.03±0.05	12019.33±6178.91
ykoK	144.26±63.44	81.06±54.94	0.65±0.38	4.74±0.45	144.26±63.44	0.81±0.20	449.03±526.67	482.97±27.04	0.00±0.00	12693.37±7330.66
Pooled Average	118.67±47.86	50.35±42.33	0.53±0.42	2.30±2.12	114.86±125.33	0.64±0.36	625.53±2554.61	556.82±227.00	0.03±0.04	5317.96±8585.19

Comparison of alignment length and positive predictive value (PPV) for *pairwise local alignment* by RNAmountAlign against the widely used local alignment software FOLDALIGN and LocARNA. Local alignment benchmarking was performed on 1500 pairwise alignments (75 alignments per family, 21 Rfam families) extracted from the Rfam 12.0 database [34], and prepared in a manner analogous to that of the dataset used in benchmarking *multiple* local alignment in [48]—see text for details. The 21 Rfam families are: RF00001, RF00002, RF00003, RF00004, RF00005, RF00008, RF00015, RF00027, RF00031, RF00050, RF00059, RF00104, RF00162, RF00167, RF00168, RF00169, RF00174, RF00181, RF00233, RF00380, RF00436. Parameters used in Eq (17) of the main text for RNAmountAlign were structural similarity weight *γ* = 1/2, gap initiation *g*_*i*_ = −3, gap extension *g*_*e*_ = −1; since reference alignments were required to have at most 70% sequence identity, nucleotide similarity matrix RIBOSUM8570-25 was used in RNAmountAlign.

Taken together, these results suggest that RNAmountAlign has comparable accuracy, but much faster run time, hence making it a potentially useful tool for genome processing applications. Here it should be stressed that all benchmarking results used equally weighted contributions of sequence and ensemble structural similarity; i.e. parameter *γ* = 1/2 when computing similarity by Eq (17).

### Statistics for pairwise alignment

**Fig 5:**
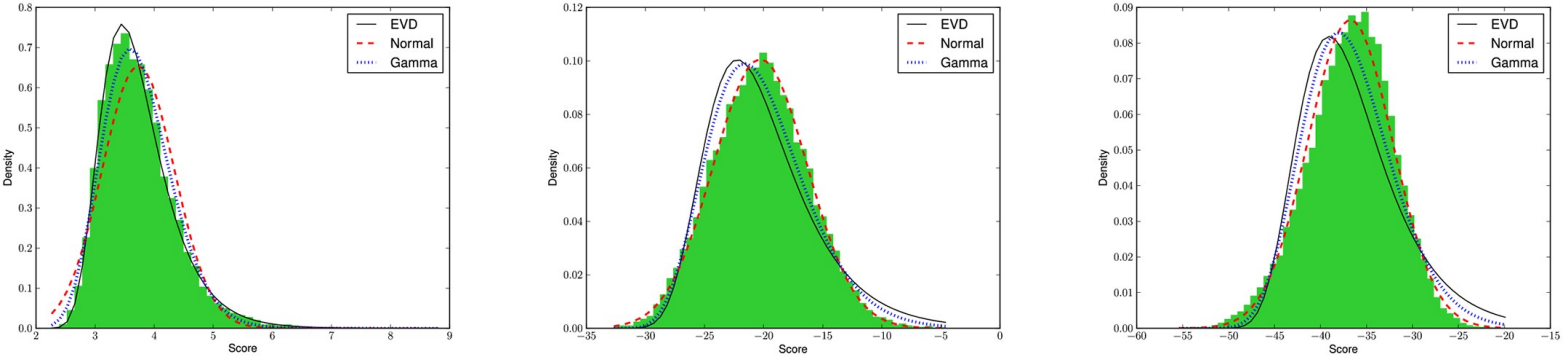
Relative frequency histograms of alignment scores for *local* (left), *semiglobal* (middle) and *global* (right) alignments of random RNAs produced by RNAmountAlign. For the 5S rRNA AY544430.1:375-465 from the Rfam 12.0 database having nucleotide relative frequencies *p*_*A*_ = 0.25, *p*_*C*_ = 0.27, *p*_*G*_ = 0.26, *p*_*U*_ = 0.21, we generated 10,000 random sequences having the same nucleotide relative frequencies, each of length 400 nt. For each method (local, semiglobal, global), RNAmountAlign was run using default parameters to determine the optimal pairwise alignment score, when aligning the 5S rRNA with each random RNA, thus producing relative frequency histograms which were subsequently fit by the normal distribution (ND), extreme value distribution (EVD) and gamma distribution (GD). As expected by Karlin-Altschul theory [12], local alignment scores are best fit by EVD, while semiglobal alignment scores are best fit by ND. Our conclusions of the best fitting distributions were additionally supported by d computations of variation distance, symmetrized Kullback-Leibler distance, and *χ*^2^ goodness-of-fit tests (data not shown).

**Fig 6:**
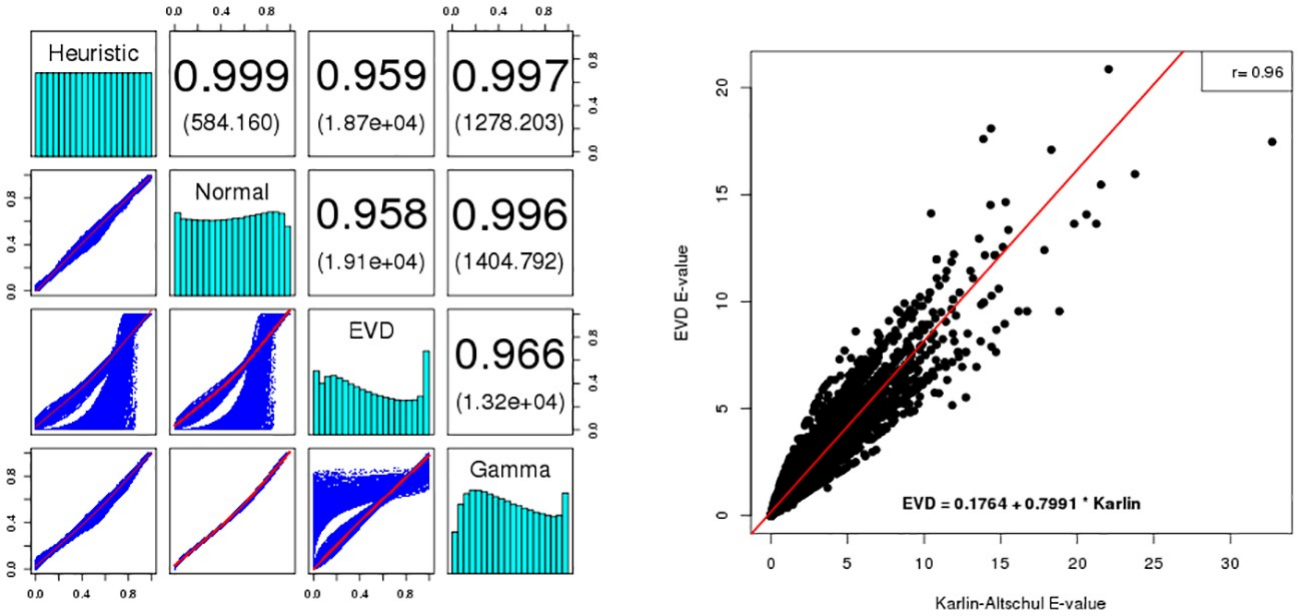
(**Left**) Pearson correlation values and scatter plots for *p*-values of *semiglobal alignment* scores between Rfam sequences and random RNAs. For 500 Rfam sequences, 1000 random semiglobal alignments were computed for 5 different gap penalties yielding the total of 2.5 million alignment scores. For each score a *p*-value is computed assuming Normal (ND), Gamma (GD) and Extreme Value (EVD) distributions in order to investigate which distribution is closest to the heuristic *p*-value, that is assumed to be the gold standard. Heuristic *p*-value for score *z* is determined by the proportion of alignment scores that exceed *z*. For all 2.5 million *p*-values, pairwise Pearson correlation values were computed and displayed in the upper triangular portion of the figure, with sums of squared residuals shown in parentheses, and histograms of *p*-values along the diagonal. It follows that ND *p*-values correlate best with heuristic *p*-values. (**Right**) Scatter plot of E-values computed by EVD fitting, *E*_EVD_, as well as our implementation of the Karlin-Altschul, *E*_KA_, for *pairwise local* alignments. The regression equation is *E*_EVD_ = 0.1764 + 0.7991 · *E*_KA_; Pearson correlation between *E*_EVD_ and *E*_KA_ is 96%, with correlation *p*-value of 2 ⋅ 10^−16^ indicating that *p*-values obtained from these two methods are in well agreement. E-values were determined from local alignment scores computed by the genome scanning form of RNAmountAlign with query tRNA AB031215.1/9125-9195 and targets consisting of 300 nt windows from *E. coli str. K-12* MG1655 with GenBank accession code NC_000913.

Fig 5 shows fits of the relative frequency histogram of alignment scores with the normal (ND), extreme value (EVD) and gamma (GD) distributions, where local, semiglobal and global alignment scores are shown in panels from left to right. The EVD provides the best fit for local alignment sequence-structure similarity scores, as expected by Karlin-Altschul theory [12, 13]. Moreover, Fig 6 shows a 96% correlation between (expect) E-values computed by our implementation of the Karlin-Altschul method, and E-values obtained by EVD fitting of local alignment scores. In contrast, the ND provides the best fit for semiglobal sequence/structure alignment similarity scores, at least for the sequence considered in Fig 5. This is not an isolated phenomenon, as shown in Fig 6, which depicts scatter plots, Pearson correlation values and sums of squared residuals (SSRs) when computing *p*-values for semiglobal alignment scores between Rfam sequences and random RNA. As explained earlier, a pool of 2220 sequences from the Rfam 12.0 database [34] was created by selecting one sequence of length at most 200 nt from each family, with the property that base pair distance between its minimum free energy (MFE) structure and the Rfam consensus structure was a minimum. Then 500 sequences were randomly selected from this pool, and for each of five gap initiation and extension costs *g*_*i*_ = −5, −4, −3, −2, −1 with $g_{e}=\frac{g_{i}}{3}$. Taking each of the 500 sequences successively as query sequence and for each choice of parameters, 1000 random 400 nt RNAs were generated with the same expected nucleotide relative frequency as that of the query. For each alignment score *z* for query and random target, the *p*-value was computed as 1 minus the cumulative density function, 1 − *CDF*(*z*), for fitted normal (ND), extreme value (EVD) and gamma (GD) distributions, thus defining 1000 *p*-values. Additionally, a heuristic *p*-value was determined by calculating the proportion of alignment scores for given query that exceed *z*. For each set of 2.5 million (500 × 5 × 1000) *p*-values (heuristic, ND, EVD, GD), Pearson correlation values were computed and displayed in the upper triangular portion of Fig 6, with SSRs shown in parentheses. Note that residuals were computed for regression equation row = *m* ⋅ column + *b*, where column values constitute the independent variable. Assuming that heuristic *p*-values constitute the reference standard, it follows that *p*-values computed from the normal distribution correlate best with semiglobal alignment scores computed by RNAmountAlign. Earlier studies have suggested that protein global alignment similarity scores using PAM120, PAM250, BLOSUM50, and BLOSUM62 matrices appear to be fit best by the gamma distribution (GD) [49], and that semiglobal RNA sequence alignment similarity scores (with no contribution from structure) appear to be best fit by GD [50]. However, in our preliminary studies (not shown), it appears that the type of distribution (ND, EVD, GD) that best fits RNAmountAlign semiglobal alignment depends on the gap costs applied (indeed, for certain choices, EVD provides the best fit). Since there is no mathematical theory concerning alignment score distribution for global or semiglobal alignments, it must be up to the user to decide which distribution provides the most reasonable *p*-values.

### Multiple alignment

**Fig 7:**
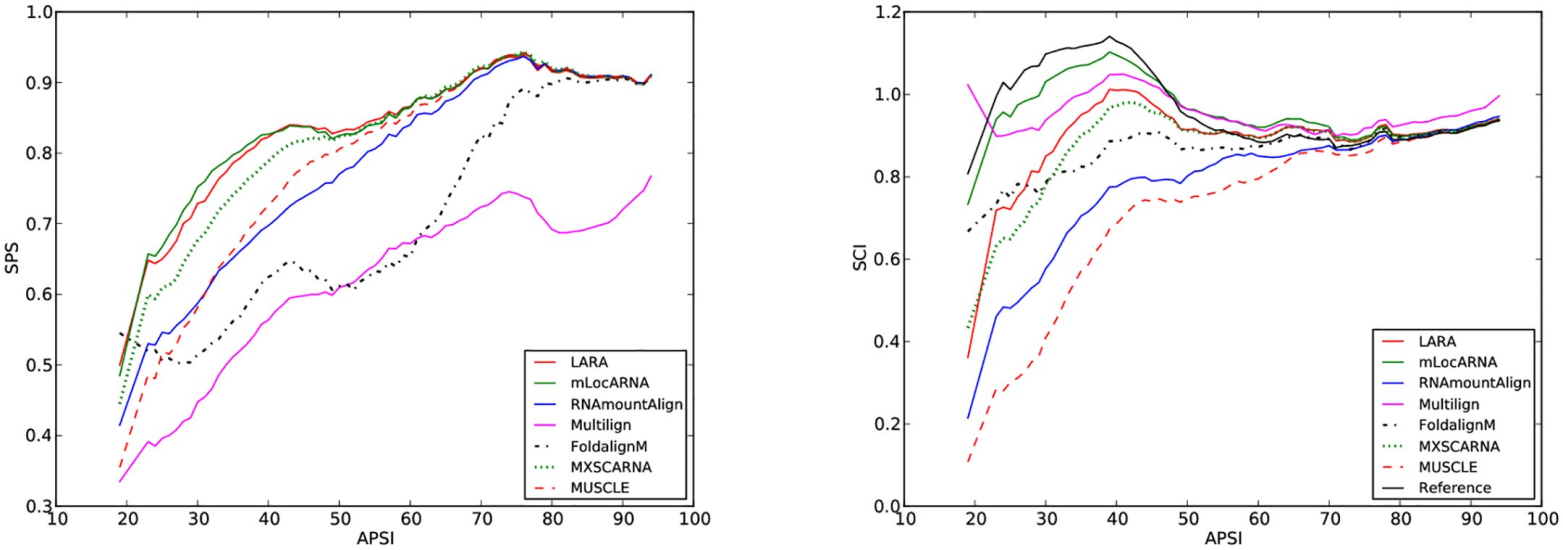
Sum-of-pairs (SPS) score (left) and structural conservation index (SCI) (right) for *multiple global alignments* using RNAmountAlign, LARA, mLocARNA, FoldalignM, Multilign, MXSCARNA and MUSCLE. SPS and SCI are shown as a function of average pairwise sequence identity(APSI). The k5 BRAliBase 2.1 database was used for benchmarking. Moving averages taken for centered, symmetric windows of size 11.

**Fig 8:**
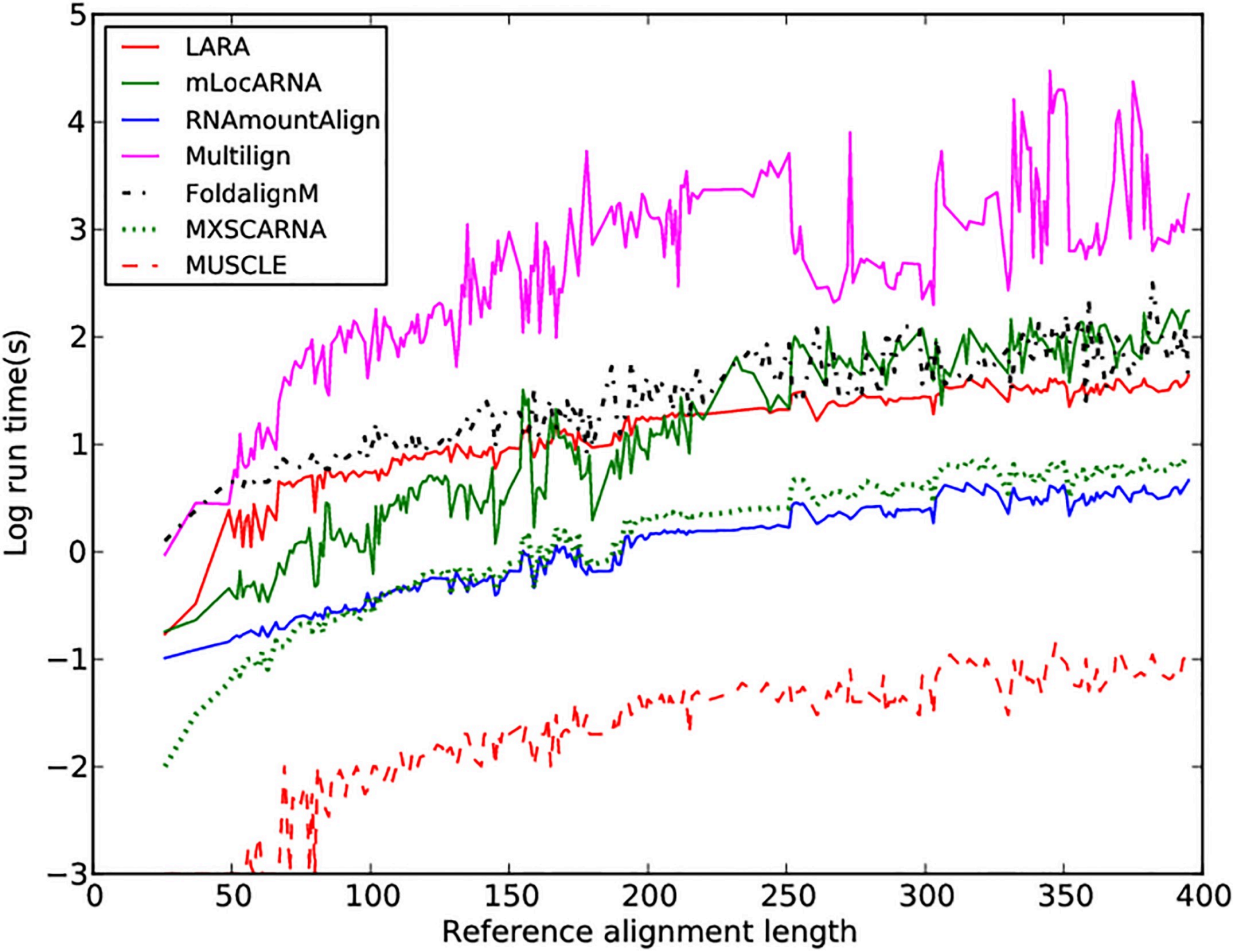
Run time of *multiple global alignment* for RNAmountAlign, mLocARNA, LARA, FoldalignM, Multilign, MXSCARNA and MUSCLE. Log run time is as shown a function of reference alignment length for K5 alignments in BRAliBase 2.1. For the structural *multiple alignment* algorithms benchmarked on this data, RNAmountAlign and MXSCARNA both appear to have the fastest runtime, while RNAmountAlign is faster than MXSCARNA for *pairwise* alignment.

We benchmarked RNAmountAlign with the software LARA, mLocARNA, FOLDALIGNM, Multilign, MXSCARNA, and sequence only MUSCLE for *multiple global* K5 alignments in BRAliBase 2.1. STRAL is not included since the source code could not be compiled. Fig 7 indicates average SPS and SCI as a function of average pairwise sequence identity (APSI). We used the -sci flag of RNAalifold to compute SCI from the output of each software without reference to the reference alignment. Fig 7 indicates that SCI values for outputs from various alignment algorithms is higher than the SCI value from reference alignments, suggesting that the consensus structure obtained from sequence/structure alignment algorithms has a larger number of base pairs than the the consensus structure obtained from reference alignments (this phenomenon was also in [51]). Fig 7 indicates that RNAmountAlign produces SPS scores comparable to mLocARNA, LARA and MXSCARNA, and higher than Multilign and FOLDALIGNM while the SCI score obtained from RNAmountAlign are lower than other software. Averaging over all sequences, the SPS scores for RNAmountAlign, LARA, mLocARNA, FOLDALIGNM, Multilign, MXSCARNA, and MUSCLE were respectively: 0.81 ± 0.18, 0.85 ± 0.15, 0.86 ± 0.15, 0.74 ± 0.24, 0.63 ± 0.17, 0.84 ± 0.15, and 0.82 ± 0.17, while SCI scores are respectively: 0.84 ± 0.24, 0.92 ± 0.22, 0.96 ± 0.21, 0.88 ± 0.23, 0.96 ± 0.21, 0.91 ± 0.20, and 0.79 ± 0.26. SPS score for each reference alignments is 1 by definition, and average SCI score over all reference alignments is 0.79 ± 0.26. Fig 8 indicates software run time in seconds on a logarithmic scale. This figure clearly shows that RNAmountAlign has faster run time than all other structural alignment software in our benchmarking tests, thus confirming the earlier result from pairwise benchmarking.

### Query scan

**Fig 9:**
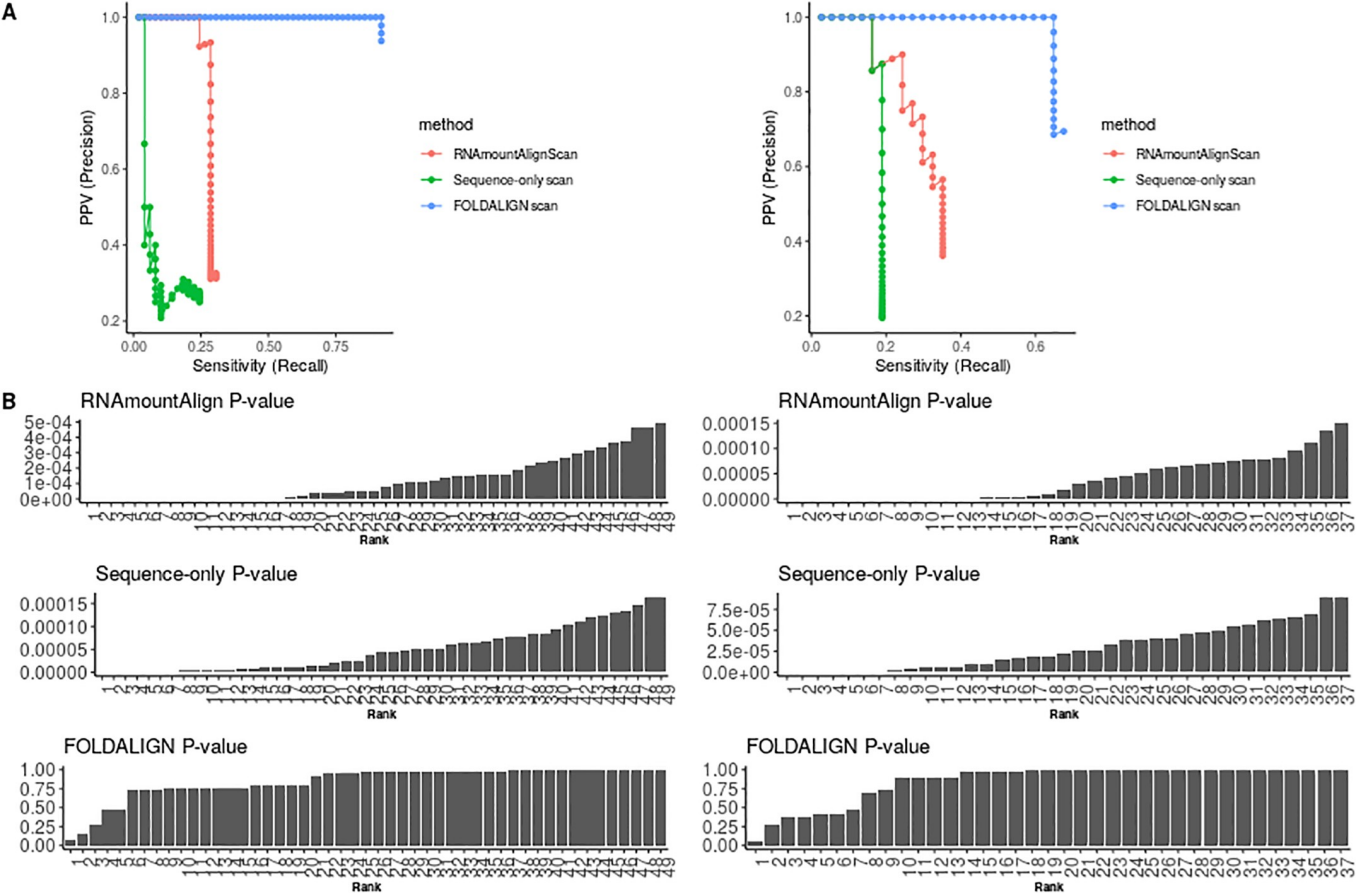
(**A**) PPV-sensitivity (precision-recall) plot for tRNA query scan in *E. coli* genome. Measures are computed for the top 49 and 37 tRNA predictions in forward (*left*) and reverse (*right*) strands of *E. coli* genome, respectively. Since RSEARCH had no true positives and thus zero sensitivity and PPV, it is not shown here. **(B)** The predicted *p*-values of the top predictions for each software in forward (*left*) and reverse (*right*) strands.

We developed an extension of RNAmountAlign, called RNAmountAlignScan, which scans a target genome sequence to find hits having high sequence and structural similarity to a given query sequence. RNAmountAlignScan functions by computing optimal semiglobal alignments of the query to sliding windows of the target, then returns the aligned target segments sorted by *p*-value. For a query of length *n*, target genome of length *m*, window size *w* > *n*, and window step size of *s*, a total of $\frac{m}{s}$ many semiglobal alignments must be computed, making the total run time of RNAmountAlignScan $O(\frac{m}{s}w^{3})$. In order to show the utility of RNAmountAlignScan, we compared it with RSEARCH [30], FOLDALIGN and RNAmountAlignScan *sequence-only* where only sequence homology is considered. RSEARCH takes a single query sequence with its secondary structure and performs local alignment with the target genome, in order to search for homologous RNA. For our comparison, we used the query sequence from Fig 6, a 71 nt tRNA from Rfam 12.0, selected with the property that the base pair distance between its MFE structure and its Rfam consensus structure is a minimum compared with other tRNAs from Rfam 12.0. As target genome, we used the *E. coli* K12 MG1655 genome having 4, 641, 652 nt. Since tRNAscan-SE [52] is generally considered to be the gold standard in tRNA prediction, we measured accuracy of the software by the amount of overlap between returned predictions and predicted tRNAs according to tRNAscan-SE. tRNAscan-SE found 49 full tRNA sequences on the forward strand and 37 on the reverse strand. Default parameters of each software package were used to search the query in the forward strand of *E. coli* genome (see S3 Table); in the case of RNAmountAlignScan this means gap initiation of −3, gap extension of −1, and sequence/structure weighting parameter *γ* = 0.5. RNAmountAlignScan was also used to scan the *E. coli* genome using sequence-only semiglobal alignments, in which gap parameters were unchanged, but with *γ* = 0. Genome scanning was performed using windows of 300 nt, with step size 200, thus ensuring an overlap of 100 nt between target segments—this resulted in a total of 23, 209 semiglobal alignments. We also tested FOLDALIGN, which provides an option to scan two sequences and return the best local alignment score for each pair of positions in the two sequences. The output from FOLDALIGN with this option then requires subsequent postprocessing using the LocateHits script included in FOLDALIGN in order to generate a list of non-overlapping local alignments. For the query tRNA described above, FOLDALIGN could not process the *E. coli* genome, and instead produced memory errors. There is a newly developed, specialized form of FOLDALIGN, called “Foldalign version 2.5.1_long_sequences” that we recently learned of from Jakob Havgaard through personal communication and can process long RNA sequences (of more than 30,000 nt) without such memory errors. Fig 9 indicates the precision-recall plot for the top 49 and 37 tRNA predictions on the forward and reverse strands, respectively. RSEARCH is not shown in the plot because it reported 89 hits, none of which had any overlap with tRNAs predicted by tRNAscan-SE and thus its sensitivity and PPV are equal to zero. Sequence length of the predictions returned by RSEARCH was 11.73 ± 0.58. For calculation of PPV as a function of sensitivity, for each of the three methods on each strand, we sorted the predictions in descending order by *p*-value. Given the *i*-th prediction, we determined the number of predictions preceding the current *i*-th prediction (including the *i*-th), and calculated the number of true positives in that collection then divided this number by 49 for positive or 37 for reverse strands to obtain sensitivity. True positives are predictions with *overlap proportion* of greater than 0.8 to any of the tRNAscan-SE sequences. The overlap proportion between predictions and tRNAscan-SE is defined as the ratio |*A* ∩ *B*|/|*B*|, where *A* is a tRNA predicted by each software and *B* is a tRNA detected by tRNAscan-SE, the latter assumed to be the gold standard. FOLDALIGN with area under the curve of 0.90 and 0.64 for forward and reverse strands outperforms RNAmountAlignScan with 0.27 and 0.28 and *sequence-only* with 0.08 and 0.16. However, only one the FOLDALIGN predictions on each strand had *p*-value less than 0.1 while RNAmountAlignScan reported significant *p*-values for all of the true positives Fig 9. Moreover, FOLDALIGN finished in 12.06 hours while run time of RNAmountAlign was 1.63.

**Fig 10:**
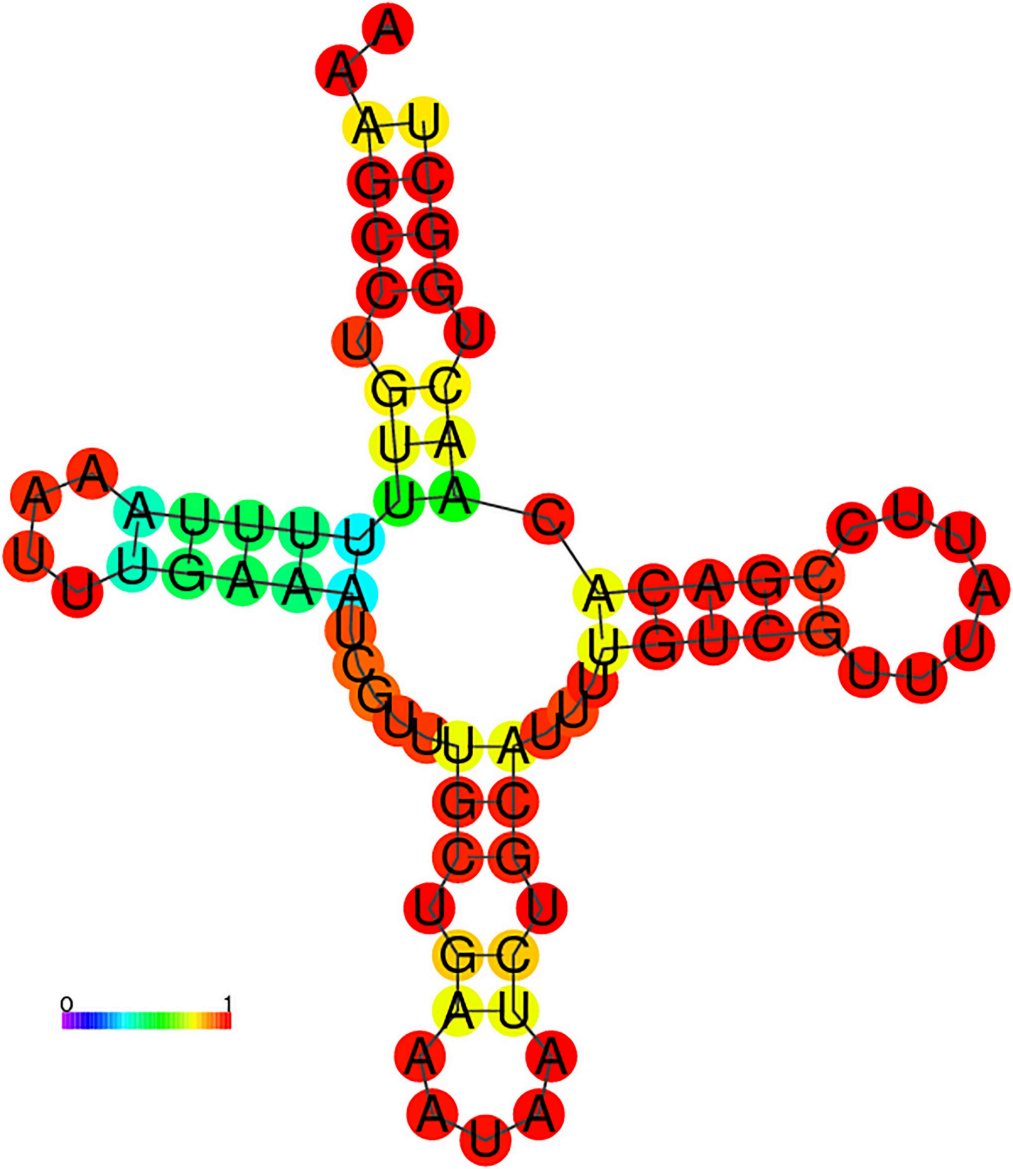
The MFE secondary structure of an *E. coli* segment which is predicted to be a tRNA by RNAmountAlignScan and not by tRNAscan-SE. Two flanking nucleotides on both ends are added to the hit sequence. The structure is color-coded by base pairing probability generated by RNAfold web server, where red [resp. blue] base pairs (*i*, *j*) have base pairing probability *p*_*i*,*j*_ ≈ 1 [resp. *p*_*i*,*j*_ ≈ 0].

Fig 10 depicts the sequence and MFE structure of the first *false positive* of RNAmountAlignScan; i.e. that prediction having statistically significant sequence and structural similarity to the query tRNA, but which shares no overlap with a tRNA predicted by tRNAscan-SE. This prediction has 42% sequence identity to the query tRNA, and its MFE forms a cloverleaf secondary structure. In contrast to all other benchmarking computations of this paper, due to the memory requirements for genome scanning mode, each program for query search was run on a 24-processor Intel Xeon CPU E5-2440 2.40 GHz system with 198GB memory. The commands to run the software are given in S3 Table. Run time for FOLDALIGN, RNAmountAlignScan, and RSEARCH were respectively 12.06, 1.63, and 0.62 hours and memory usage were 0.57, 20.61 and 0.49 Gigabytes. The number of random RNAs used for *p*-value computation in both RSEARCH and RNAmountAlignScan was 1000, and both used the RIBOSUM 85-60 matrix. The outputs of all the program are available at http://bioinformatics.bc.edu/clotelab/RNAmountAlign/.

## Conclusion

RNAmountAlign is a new C++ software package for RNA local, global, and semiglobal sequence/structure alignment, which provides accuracy comparable with that of a number of widely used programs, but provides much faster run time. RNAmountAlign additionally computes E-values for local alignments, using Karlin-Altschul statistics, as well as *p*-values for normal, extreme value and gamma distributions by parameter fitting.

## Supporting information

S1 Table Commands used for benchmarking various software packages for global alignment. (TIFF)

S2 Table Commands used for benchmarking various software packages for local alignment. (TIFF)

S3 Table Commands used for benchmarking software packages for query scan. (TIFF)

S4 Table *p*-values from two-tailed paired Wilcoxon signed rank test of all 8,976 F1 scores for pairwise global alignments indicated in Table 4 of the main text. Tables of *p*-values computed separately for each family are available at http://bioinformatics.bc.edu/clotelab/RNAmountAlign.
(TIFF)

S5 Table *p*-values from two-tailed paired Wilcoxon signed rank test of all 1500 positive predictive values for pairwise local alignments indicated in Table 5 of the main text. Tables of *p*-values computed separately for each family are available at http://bioinformatics.bc.edu/clotelab/RNAmountAlign.
(TIFF)

S6 Table Average sensitivity scores (± one standard deviation) for *pairwise global alignment* of RNAmountAlign and four widely used RNA sequence/structure alignment algorithms on the benchmarking set of 8,976 pairwise alignments from the BRaliBase K2 database [38]. For each indicated Rfam family, the the number of alignments (NumAln), sequence identity (SeqId), and sensitivity scores for RNAmountAlign, LocARNA, LARA, FOLDALIGN, DYNALIGN, STRAL and MXSCARNA are listed, along with pooled averages over all 8,976 pairwise alignments. Parameters used in Eq (17) of the main text for RNAmountAlign were similarity matrix RIBOSUM85-60, structural similarity weight *γ* = 1/2, gap initiation *g*_*i*_ = −3, gap extension *g*_*e*_ = −1.
(TIFF)

S7 Table Average positive predictive value (PPV) scores (± one standard deviation) for *pairwise global* alignment of RNAmountAlign and four widely used RNA sequence/structure alignment algorithms on the benchmarking set of 8,976 pairwise alignments from the BRaliBase K2 database [38]. For each indicated Rfam family, the the number of alignments (NumAln), sequence identity (SeqId), and PPV-scores for RNAmountAlign, LocARNA, LARA, FOLDALIGN, DYNALIGN, STRAL and MXSCARNA are listed, along with Pooled averages over all 8,976 pairwise alignments. Parameters used in Eq (17) of the main text for RNAmountAlign were similarity matrix RIBOSUM85-60, structural similarity weight *γ* = 1/2, gap initiation *g*_*i*_ = −3, gap extension *g*_*e*_ = −1.
(TIFF)

S8 TableInitial portion of a table that determines expected base pairing probabilities *p*_(_, *p*_•_, *p*_)_ as a function of nucleotide probabilities *p_A_*, *p_C_*, *p_G_*, *p_U_*.The full table (not shown) has 1770 rows. To determine average base pairing probabilities, given nucleotide probabilities *p_A_*, *p_C_*, *p_G_*, *p_U_*, a total of *N* = 10000 RNA sequences of length *n* = 200 were randomly generated to have the given expected nucleotide frequency. To compute *p*_(_ [resp. *std*_(_], a library call of function pf_fold() from Vienna RNA Package [4] was made in order to determine $Prob\left[i\;\text{pairs}\;\text{to}\;\text{right}\right]=\sum_{i=1}^{n}\sum_{j=i+1}^{n}p_{i,j}$ for position in each sequence, and the average [resp. standard deviation] was taken over all sequences and values *i* = 1, …, *n*. In a similar fashion, *p*_•_ and *p*_)_ were determined.(TIFF)

S1 Fig Consensus structure for the pairwise alignment indicated in Fig 1 of the main text. The consensus structure is computed by a calling function alifold() from Vienna RNA Package. The figure is obtained from RNAalifold web server.
(TIFF)

S2 Fig F1-score for RNAmountAlign, LocARNA, LARA, FOLDALIGN, DYNALIGN, STRAL, MXSCARNA and sequence-only alignments(*γ* = 0) for *pairwise global alignment*. Moving averages of F1-score for centered, symmetric windows of size 11 are shown as a function of sequence identity for pairwise alignments in the BRAliBase 2.1 database used for benchmarking. Moving averages taken for centered, symmetric windows of size 11.
(TIFF)

S3 Fig Average sensitivity (Sen) and positive predictive value (PPV) for RNAmountAlign, LocARNA, LARA, FOLDALIGN, DYNALIGN, STRAL, MXSCARNA and sequence-only alignments (*γ* = 0) for *pairwise global alignment*. Sensitivity is shown as a function of sequence identity for pairwise alignments in the BRAliBase 2.1 database used for benchmarking. Moving averages taken for centered, symmetric windows of size 11.
(TIFF)

S4 Fig Average pairwise sensitivity (left) and positive predictive value (right) for *multiple global alignments* using RNAmountAlign, LARA, mLocARNA, FoldalignM and Multilign in the k5 BRAliBase 2.1 database used for benchmarking. Note that in our definition of *Sen* and *PPV*, pairs of the form (*X*, —) and (—, *X*) are also counted while SPS is the average pairwise sensitivity only considering aligned residue pairs (Fig 7). However, the results with and without gap counts, indicated in this figure and Fig 7, respectively, are very close. Moving averages taken for centered, symmetric windows of size 11.
(TIFF)

S5 Fig Matthews Correlation Coefficient (MCC) for the quality of secondary structure prediction (see text) from each of RNAmountAlign, LocARNA, LARA, FOLDALIGN, DYNALIGN, STRAL, MXSCARNA and sequence-only alignments (*γ* = 0) for *pairwise global alignment*. Moving averages of MCC are computed for centered, symmetric windows of size 11 and shown as a function of sequence identity for a subset of 7, 154 reference pairwise alignments from k2 BRAliBase 2.1. Overall average MCC values ± one standard deviation are shown in parentheses. These 7, 154 alignments were selected with the property that both sequences in the alignment (exactly) appear in an Rfam family seed multiple alignment from Rfam 7.0, and so can be assigned an Rfam consensus secondary structure as described in the text. These consensus structures are taken as the reference structures in the computation of MCC. Predicted structures are obtained directly from the output of each software in the benchmarking test. For RNAmountAlign and for our in-house implementation of STRAL, the -alifold flag was used to compute the consensus structure by a function call to alifold() from libRNA.a in the Vienna RNA Package.
(TIFF)

S6 Fig F1-score for RNAmountAlign, LocARNA, LARA, FOLDALIGN, DYNALIGN, STRAL, MXSCARNA and sequence-only alignments(*γ* = 0) for *pairwise global alignment*, where any failure of the benchmarked program to output an assignment is simply ignored. Moving averages of F1-score for centered, symmetric windows of size 11 are shown as a function of sequence identity for pairwise alignments in the BRAliBase 2.1 database used for benchmarking. Moving averages taken for centered, symmetric windows of size 11. This figure should be compared with S2 Fig, where failure to output an alignment is counted as zero.
(TIFF)

S7 Fig Average sensitivity (Sen) and positive predictive value (PPV) for RNAmountAlign, LocARNA, LARA, FOLDALIGN, DYNALIGN, STRAL, MXSCARNA and sequence-only alignments (*γ* = 0) for *pairwise global alignment*, where any failure of the benchmarked program to output an assignment is simply ignored. Sensitivity is shown as a function of sequence identity for pairwise alignments in the BRAliBase 2.1 database used for benchmarking. Moving averages taken for centered, symmetric windows of size 11. This figure should be compared with S3 Fig, where failure to output an alignment is counted as zero.
(TIFF)

S8 Fig Illustration of a potential weakness of RNAmountAlign. Using RNAmountAlign genome-scanning software, semiglobal alignments of the query tRNA AB031215.1/9125-9195 were made with each 300 nt window (successive window overlap of 200 nt) of the *E. coli* str. K-12 substr. MG1655 genome. This figure shows the MFE structure, color-coded by positional entropy [53], for the alignment of positions 696097-696164 with score −7.70, *p*-value of 4.145010 ⋅ 10^−6^. (gap costs *g*_*i*_ = −3, *g*_*i*_ = −1, *γ* = 0.5, scaling factor *α*_seq_ = 0.447648, shift term *α*_str_ = 0.304766, *γ* = 1/2). However, this RNA is clearly not a tRNA, since the three loops are not within the scope of a multiloop, and the variable loop is located in the wrong position, and the large positional entropy suggests that there is not an unambiguous structure. Moreover, this sequence is not one of the 40 tRNA genes/pseudogenes on the plus-strand predicted by tRNAscan-SE [52].
(TIFF)

S1 Appendix Software tutorial. (PDF)
