# Supplementary figures and images for: RNAmountAlign: Efficient software for local, global, semiglobal pairwise and multiple RNA sequence/structure alignment
Source: PLoS One. 2020 Jan 24;15(1):e0227177. doi: 10.1371/journal.pone.0227177 (PMC6980424; doi:10.1371/journal.pone.0227177)

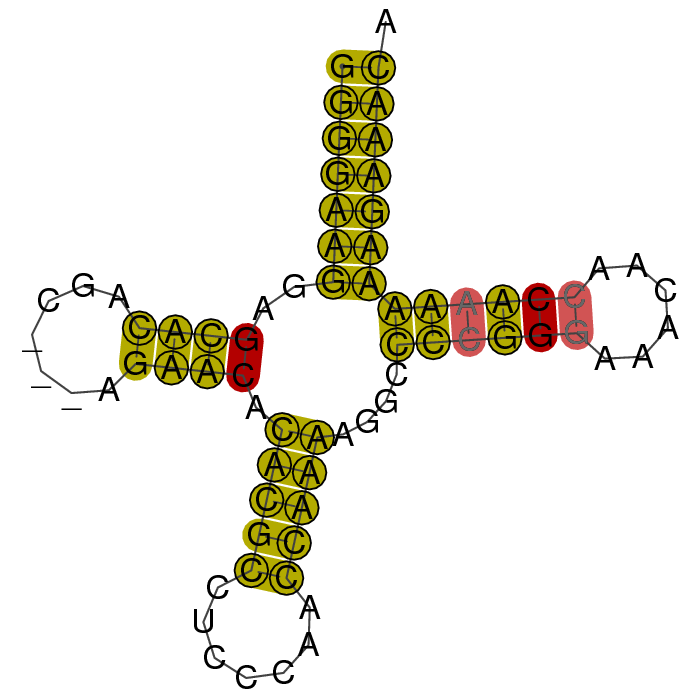

Supplement: S1 Fig — The consensus structure is computed by a calling function alifold() from Vienna RNA Package. The figure is obtained from RNAalifold web server. (TIFF) [file pone.0227177.s009.tiff]

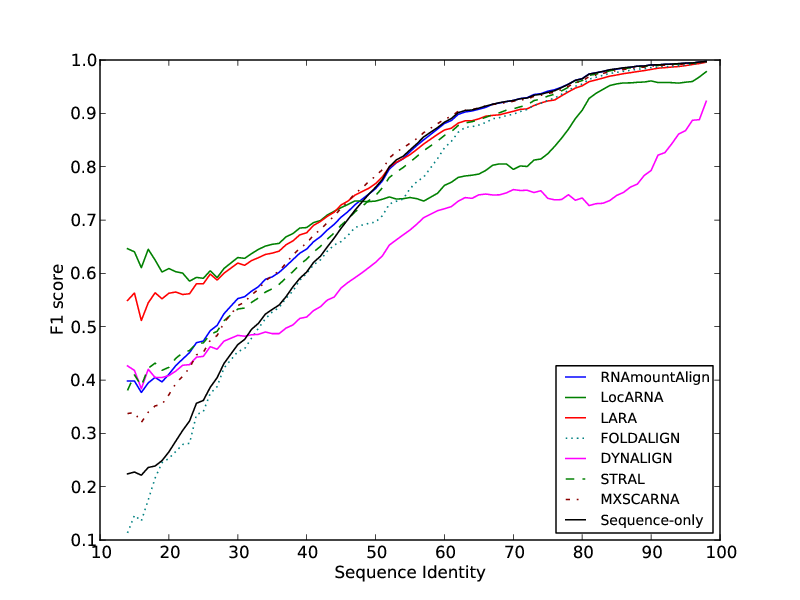

Supplement: S2 Fig — Moving averages of F1-score for centered, symmetric windows of size 11 are shown as a function of sequence identity for pairwise alignments in the BRAliBase 2.1 database used for benchmarking. Moving averages taken for centered, symmetric windows of size 11. (TIFF) [file pone.0227177.s010.tiff]

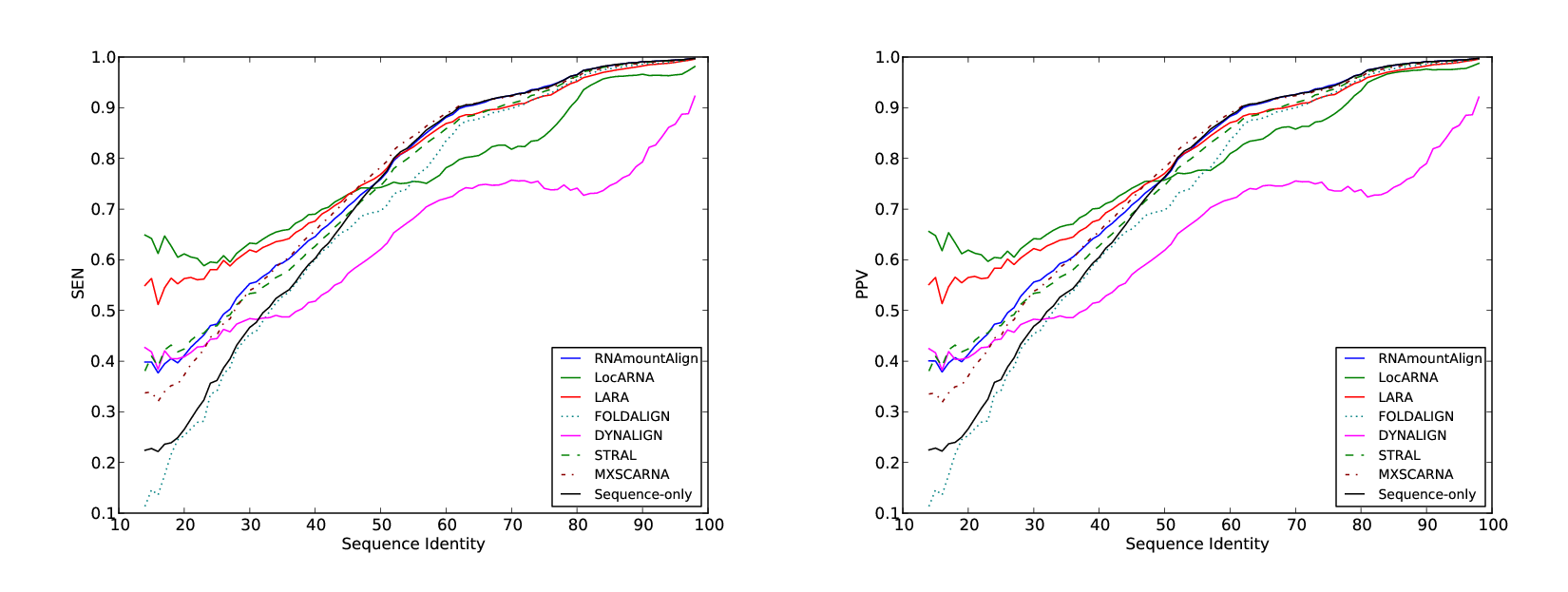

Supplement: S3 Fig — Sensitivity is shown as a function of sequence identity for pairwise alignments in the BRAliBase 2.1 database used for benchmarking. Moving averages taken for centered, symmetric windows of size 11. (TIFF) [file pone.0227177.s011.tiff]

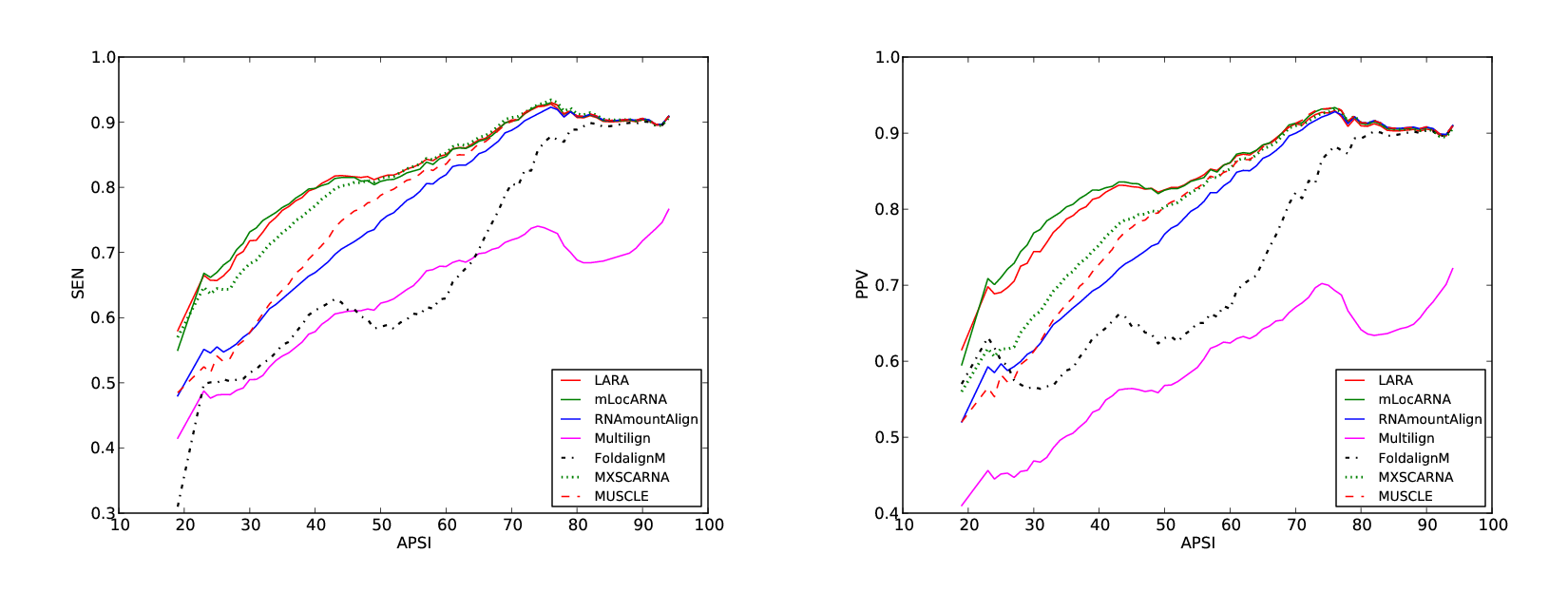

Supplement: S4 Fig — Note that in our definition of Sen and PPV, pairs of the form (X, —) and (—, X) are also counted while SPS is the average pairwise sensitivity only considering aligned residue pairs (Fig 7). However, the results with and without gap counts, indicated in this figure and Fig 7, respectively, are very close. Moving averages taken for centered, symmetric windows of size 11. (TIFF) [file pone.0227177.s012.tiff]

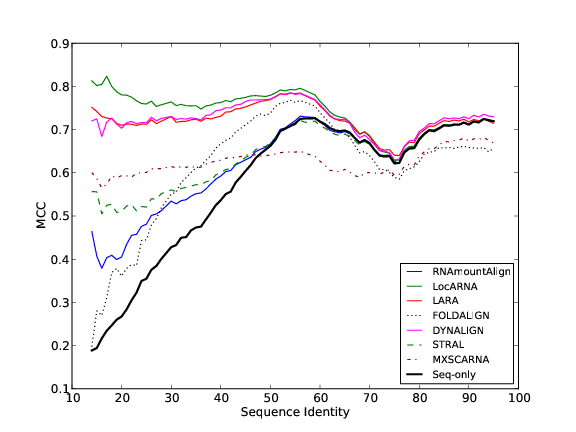

Supplement: S5 Fig — Moving averages of MCC are computed for centered, symmetric windows of size 11 and shown as a function of sequence identity for a subset of 7, 154 reference pairwise alignments from k2 BRAliBase 2.1. Overall average MCC values ± one standard deviation are shown in parentheses. These 7, 154 alignments were selected with the property that both sequences in the alignment (exactly) appear in an Rfam family seed multiple alignment from Rfam 7.0, and so can be assigned an Rfam consensus secondary structure as described in the text. These consensus structures are taken as the reference structures in the computation of MCC. Predicted structures are obtained directly from the output of each software in the benchmarking test. For RNAmountAlign and for our in-house implementation of STRAL, the -alifold flag was used to compute the consensus structure by a function call to alifold() from libRNA.a in the Vienna RNA Package. (TIFF) [file pone.0227177.s013.tiff]

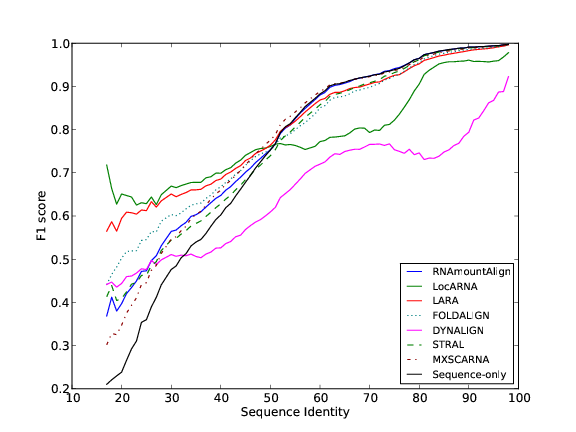

Supplement: S6 Fig — Moving averages of F1-score for centered, symmetric windows of size 11 are shown as a function of sequence identity for pairwise alignments in the BRAliBase 2.1 database used for benchmarking. Moving averages taken for centered, symmetric windows of size 11. This figure should be compared with S2 Fig, where failure to output an alignment is counted as zero. (TIFF) [file pone.0227177.s014.tiff]

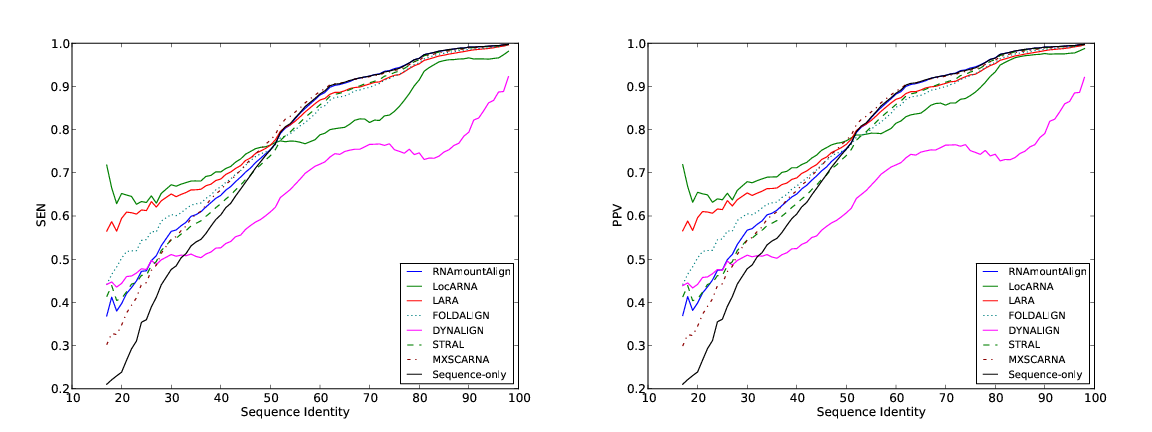

Supplement: S7 Fig — Sensitivity is shown as a function of sequence identity for pairwise alignments in the BRAliBase 2.1 database used for benchmarking. Moving averages taken for centered, symmetric windows of size 11. This figure should be compared with S3 Fig, where failure to output an alignment is counted as zero. (TIFF) [file pone.0227177.s015.tiff]

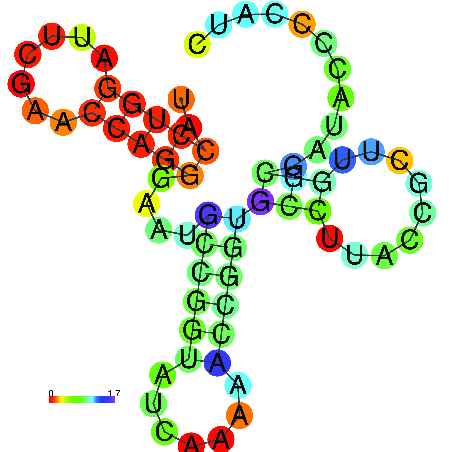

Supplement: S8 Fig — Using RNAmountAlign genome-scanning software, semiglobal alignments of the query tRNA AB031215.1/9125-9195 were made with each 300 nt window (successive window overlap of 200 nt) of the E. coli str. K-12 substr. MG1655 genome. This figure shows the MFE structure, color-coded by positional entropy [53], for the alignment of positions 696097-696164 with score −7.70, p-value of 4.145010 ⋅ 10−6. (gap costs gi = −3, gi = −1, γ = 0.5, scaling factor αseq = 0.447648, shift term αstr = 0.304766, γ = 1/2). However, this RNA is clearly not a tRNA, since the three loops are not within the scope of a multiloop, and the variable loop is located in the wrong position, and the large positional entropy suggests that there is not an unambiguous structure. Moreover, this sequence is not one of the 40 tRNA genes/pseudogenes on the plus-strand predicted by tRNAscan-SE [52]. (TIFF) [file pone.0227177.s016.tiff]
