# Supplementary material for: RNAmountAlign: Efficient software for local, global, semiglobal pairwise and multiple RNA sequence/structure alignment
Source: PLoS One. 2020 Jan 24;15(1):e0227177. doi: 10.1371/journal.pone.0227177 (PMC6980424; doi:10.1371/journal.pone.0227177)
Supplement: S1 Appendix — (PDF) [file pone.0227177.s017.pdf]

Appendix S1. Software usage

RNAmountAlign performs local, semiglobal, and global sequence/structure alignments. By default the global alignment is computed unless flags -local or -semi are used to perform local and semiglobal alignments, respectively. In the simplest case, the program could be run with

```
> ./RNAmountAlign -f <inputFasta>
or
> ./RNAmountAlign -s seq1 seq2
```

The parameters that were used to produce the results in the main text are used as the default by the software: structural similarity weight $\gamma = 0.5$, gap initiation $g_i = -3$, and gap extension $g_e = -1$. The weight factor γ defines the importance of structural similarity versus sequence similarity. When $\gamma = 0$ only sequence similarity is considered, while $\gamma = 1$ only uses the incremental ensemble mountain heights for the alignment. As an example, let's consider the following two toy sequences each forming a stem loop secondary structure

```
>seq1
AAAAAAAAAACCCCCUUUUUUUUUU
((((((((((.....)))))))))) (-2.1)
>seq2
CCCCCCCAAAAGGGGGGG
(((((((....))))))) (-15.7)
```

Running the software considering only sequence similarity with gap initiation and extension penalties of -2 and -1, respectively, by the command

```
> ./RNAmountAlign -s AAAAAAAAAACCCCCUUUUUUUUUU CCCCCCCAAAAGGGGGGG -gamma 0 -gi
    -2 -ge -1
```

produces the following alignment

```
seq1  1  AAAAAAAAAACCCCCUUUUUUUUUU 25
seq2  1  -------CCCCCCCAAAAGGGGGGG 18
```

where four C nucleotides are aligned together, regardless of the fact that in the secondary structure for the first sequence, they are found in an apical loop region, while in the secondary structure for the second sequence, they are part of a stem. However, using -gamma 1 returns

```
seq1  1  AAAAAAAAAACCCCCUUUUUUUUUU 25
seq2  1  CCCCCCC----AAAAGGGGGGG--- 18
```

where the opening, closing and unpaired bases are aligned to each other. Finally, using -gamma 0.5 gives

```
seq1  1  AAAAAAAAAACCCCCUUUUUUUUUU 25
seq2  1  CCCCCCCAAAA-------GGGGGGG 18
```

where both sequence and structural similarity are equally weighted.

By setting $\gamma = 1$, RNAmountAlign alignments depend wholly on structural similarity (see Fig 1). Indeed, for the following BRAliBase 2.1 alignment with 28% sequence

identity, by setting $\gamma = 1$, `RNAmountAlign` returns the correct alignment.

```
GGGGAUGUAGCUCAGUGGUAGAGCGCAUGCUUCGCAUGUAUGAGGCCCCGGGUUCGAUCCCCGGCAUCUCCA
GUUUCAUGAGUAUAGC---AGUACAUUCGGCUUCCAACCGAAAGGUUUUUGUAAACAACCAAAAAUGAAAUA
```

of 72 nt tRNA AL671879.2 with 69 nt tRNA D16387.1. Fig 1 shows the superimposed mountain heights for this alignment.

The default nucleotide similarity matrix is RIBOSUM85-60. Other RIBOSUM matrices are included in the software and can be selected with `-m` flag based on the user's knowledge of divergence of the input sequences.

`RNAmountAlign` computes the consensus secondary structure by calling `alifold()` function from `libRNA.a` in the Vienna RNA Package when flag `-alifold` is used. For example the following command outputs the consensus structure in addition to the alignment for the same sequences indicated in Fig 1 of the main text.

```
> ./RNAmountAlign -f examples/trna.fa -alifold -global
```

Computation of alignment statistics depends on the alignment type. As discussed in the main text, local alignment scores follow extreme value distribution(EVD) while global and semiglobal scores tend to follow normal distribution(ND). Flag `-stat` can be set to compute both E-values and p-values, where the transformation between E-values and p-values is made by $p = 1 - exp(-E)$. For global and semiglobal alignments, the first (query) sequence is aligned to a number of random RNAs, defined by `-num` flag, with the same nucleotide composition as the second sequence (target), then the random alignment scores are fitted to normal distribution and a p-value is returned.

```
> ./RNAmountAlign -f examples/trna.fa -global -stat -num 100
```

As part of the output, p-value from ND normal fitting of 100 random alignment scores is reported:

```
Normal distribution E-value: 0.0476148
Normal distribution p-value: 0.046499
```

For local alignments either Karlin-Altschul statistics (default) or EVD fitting can be computed. Let's consider an example of a local alignment between two purine riboswitches with Rfam seed alignment length of 102 and sequence identity 0.58. Random flanking regions with the same nucleotide composition are added to the seed alignment as discussed in the main text to obtain two sequences of length 408 and 400. The local alignment between these two sequences has length 53 with extremely low E-value, with the property that all pairs in the local alignment are found in the reference seed alignment ($PPV = 1$). E-value from Karlin-Altschul statistics can be obtained very fast from the following command:

```
> ./RNAmountAlign -f examples/RF00167_1.raw -local -stat
Karlin-Altschul E-value: 2.52137e-06
Karlin-Altschul p-value: 2.52137e-06
```

Computation of E-value from EVD fitting is more accurate but slower:

```
> ./RNAmountAlign -f examples/RF00167_1.raw -local -stat -evd -num 200
Extreme value distribution E-value: 4.41417e-05
Extreme value distribution P-value: 4.41408e-05
```

`RNAmountAlign` computes Karlin-Altschul E-values from maximum likelihood method described in the main text, and then multiplies it by the regression coefficient of 0.7991, indicated in the right panel of Fig 6, to obtain an estimated E-value. Therefore, there

might be discrepancy between the EVD fitting and Karlin-Altschul E-values. For the most accurate statistics EVD fitting is recommended.

Our software could also be used for searching a query sequence defined by `-qf <fastaFile>` in a target sequence defined by `-tf <fastaFile>`. The search computes semiglobal alignments of the query to sliding windows of the target, and returns the aligned segments of the target sorted by p-value. The query is aligned to windows of a fixed size defined by `-window`, sliding by steps defined by `-step` flag. To compute the statistics, random alignment scores are computed and fitted to ND. However, the software does not compute random alignments for each window separately as it would be very slow. Instead, following [30], the range of the GC-content of the target sequence over all the sliding windows is first obtained and binned using bin size defined by `-gc`. For each GC-content bin, fitting parameters are precomputed by generating a number of random sequences whose GC-content is equal to the bin midpoint, aligning the query to random sequences, and fitting random alignment scores to normal distribution. For each sliding window the corresponding precomputed parameters are used for the computation of p-value. As an example, a random tRNA from Rfam 12.0 whose minimum free energy structure has the minimum base pair distance to the Rfam consensus structure was selected and used as the query to search *E. coli* K12 MG1655 genome using window size 300 and step size 200 by the following command.

```
> ./RNAmountAlignScan -qf examples/tRNAscan.fa -tf examples/ecoli_MG1655.fa
    -window 300 -step 200 -gc 10 -num 1000
```

The output contains:

```
GC Bins: [0.23-0.33),[0.33-0.43),[0.43-0.53),[0.53-0.63),[0.63-0.73),[0.73-0.74]
1000 random seqs of size 300 generated for each each GC bin.

Fitting to Normal:

  GC_Content   Location_Param   Scale_Param
  0.283        -12.18           1.96
  0.383        -13.41           2.03
  0.483        -15.01           2.05
  0.583        -16.84           2.05
  0.683        -18.98           2.16
  0.735        -20.08           2.06
```

As indicated, six GC bins are generate in range $[0.23 - 0.74]$; for each bin 1000 random sequences whose GC-content are equal to the average GC-content of the bins are generated, aligned to the query and their fitted location (mean) and scale (standard deviation) parameters are precomputed to be used for computation of p-values. From the top 20 hits of our software, the first 18 are reported to be tRNAs by `tRNAscan-SE`.

To see all the full parameter list for the software please use

```
> ./RNAmountAlign -h
```
